# Longitudinal changes in retinal image quality in school-aged children

**DOI:** 10.1364/BOE.587977

**Published:** 2026-07-02

**Authors:** Augusto Arias, Susanna P. Clement, Siegfried Wahl, Fuensanta A. Vera-Diaz

**Affiliations:** 1 ZEISS Vision Science Lab, University of Tübingen, Tübingen, Germany; 2 Carl Zeiss Vision International GmbH, Aalen, Germany; 3 Children’s Vision Lab, New England College of Optometry, Boston, MA, USA

## Abstract

Designing more effective myopia control optical-based therapies requires a better understanding of the longitudinal changes in retinal image quality across the central and peripheral retina of children with different refractive profiles. To this end, we computed through-focus image quality descriptors from ocular aberrations measured across an eccentricity range of 60°, with data acquired biannually over three years in 62 children with initial functional emmetropia participating in the PICNIC study. Our results reveal distinct spatiotemporal optical signatures associated with myopia development: longer peripheral depth of focus and reduced image quality at the near-temporal and central retina even after foveal correction, and placement of the sharpest images behind the temporal retina.

## 1. Introduction

Normal emmetropization occurs when the typical eye elongation is balanced with changes in the optics of the eye during ocular growth in childhood [1]. Although the specific mechanisms for successful emmetropization are not known, it is known that the retinal images’ properties [2], as well as the visual environmental [3] and hereditary factors [4] play a role. If successful, the emmetropization process results in an eye with near-zero refractive error, in which, when accommodation is relaxed, the retina coincides with the image focal plane for objects at infinity. If unsuccessful, the eye becomes myopic or hyperopic when the central retina lies posterior or anterior to this focal plane, respectively. In recent years, attention has been directed toward the development and treatment of myopia [5], which is generally caused by excessive axial elongation and leads to higher risk of potentially blinding ocular diseases in adulthood [6]. Additionally, the prevalence of myopia is steadily increasing, with projections suggesting that by 2050, half of the world’s population will be affected [7].

A comprehensive understanding of ocular and visual factors that disrupt normal refractive development and emmetropization is imperative for developing preventive strategies and effective treatments for myopia progression. The longitudinal study PICNIC [8] (Preventing myopia: Investigating Contributing Factors to Nearsightness in Children) was conducted for this purpose. This study enrolled 102 school-aged children with functional emmetropia, evenly divided at baseline into high- and low-risk groups for myopia. Over the 3-year follow-up period, participants underwent biannual assessments of cycloplegic refraction (measured with an open-field autorefractor), optical biometry, ocular health, and central and peripheral aberrations. The longitudinal changes in aberrations have been previously reported, showing differences in relative peripheral refraction, in the amount of high-order aberrations, primary spherical aberration (SA), and horizontal coma between children at low and high risk for myopia, as well as those who developed myopia at the end of the study [9]. This published report did not present the distribution of image quality in front of and behind the retina, which is likely important for controlling eye elongation, since most current optical interventions for myopia control aim to shift this image quality distribution anterior to the retina [10] or, hypothetically, to extend the depth of focus (DOF) [11].

The current work evaluates, for the first time, the longitudinal changes in the distribution of quality of stimuli focused in front of, on and behind the central and peripheral retina in school-aged children. This distribution, computed from central and peripheral ocular aberrations and referred to as ‘through-focus image quality’, was characterized by four parameters: the depth of focus (DOF), the overall maximum of Strehl ratio (MaxSR), the center of mass (COM), and the optical anisotropy at the retinal plane (OAn). Baseline values and three-year changes in each of these parameters were compared across groups of eyes with different refractive development patterns to evaluate their potential role in myopia development. In particular, this study examined two hypotheses of optical signaling in myopia development: the light-signal dilution hypothesis and the directional bias hypothesis. The light-signal dilution hypothesis proposes that uncontrolled axial elongation is associated with both degradation and broadening of the through-focus image quality [12]. Accordingly, this hypothesis was evaluated using DOF- and MaxSR-based analyses. On the other hand, the directional bias hypothesis suggests that refractive development is influenced by a predominant concentration of higher-quality images at positions anterior or posterior to the retina [13]. This hypothesis was therefore investigated through a COM-based analysis. In addition, OAn-based analysis was performed to assess eccentricity-dependent changes in blur orientation associated with myopia development.

## 2. Methods

### 2.1. Parametrization of the through-focus image quality

Through-focus image quality parameters were calculated from central and peripheral aberrations data from the PICNIC study. Ocular aberrations were recorded from the right eye without cycloplegia or refractive correction while the participant viewed a green light at 6 m distance. Aberrations were measured using an instrument that scans retinal eccentricities (ε) from -30° to 30° along the horizontal meridian in less than 10 seconds using an infrared beam (wavelength, λ =780 nm) [14]. At each ε, the first 21 Zernike coefficients were computed for a 4-mm pupil diameter ( ∅ ), with peripheral pupil ellipticity accounted for using the LC method (i.e., a large circular aperture enclosing the elliptical pupil) [15]. Measurements were obtained with an angular resolution of 1°. A smoothing procedure was applied to reduce measurement variability in the Zernike coefficient profiles across ε. Specifically, the coefficients were averaged pairwise over the ranges −30° ≤ ε ≤ −1° and 1° ≤ ε ≤ 30°, thereby reducing the sampling to every second half-degree, while preserving the central value at ε = 0° unchanged. To calculate off-axis image quality accurately, aberrations were reconstructed by rescaling the Zernike polynomials over an elliptical pupil with horizontal and vertical lengths of ∅ cos(ε) and ∅ , respectively.

For each ε, the through-focus image quality was quantified via the through-focus volume under the modulation-transfer-function (TF-VUMTF). The TF-VUMTF was computed by numerically propagating pupils incorporating phase maps with the ocular aberrations – reconstructed from the Zernike coefficients – and adding different amounts of defocus between -5 and +5 D. The defocus corresponded to phase maps with a radial profile given by:

(1) $$\text{Defocus = }(\pi /\lambda )D_{add}r^{2}$$

where *r* is the radial coordinate at the pupil plane, and $D_{add}$ is the amount of added defocus. The propagation of the light from the pupil for each $D_{add}$ value was calculated using the Fast Fourier transform [16], yielding the point-spread-function (PSF). Subsequently, the modulation-transfer-function (MTF) was assessed by applying the Fast Fourier transform to the PSF. The VUMTF corresponded to the integrated MTF values up to 60 cycles per degree (cpd), mathematically:

(2) $$\text{VUMTF = }\overset{N}{\underset{j=1}{\sum }}\overset{N}{\underset{i=1}{\sum }}MTF(i,j)circ(i,j)\;\Delta f^{2}$$

where MTF is the N × N matrix with indices *i* and *j* containing the MTF values, Δf is the pixel size of the MTF matrix in spatial frequency units. In this study, Δf was equal to 0.17 cpd. circ is a function for filtering out the VUMTF values for spatial frequencies higher than 60 cpd:

(3) $$\text{circ}(i,j)\text{ = }\left\{ \begin{array}{cc} 1 & for\;\Delta f\sqrt{{\left(i-\frac{N}{2}\right)}^{2}+{\left(j-\frac{N}{2}\right)}^{2}}\leq 60\;cpd\\ 0 & otherwise \end{array}\right.$$

The VUMTF was then normalized to that value for a diffraction-limited PSF (i.e., the corresponding PSF to a flat phase map). This normalized value is equivalent to the Strehl ratio. Lastly, the TF-VUMTF was computed as the list of the VUMTF for all the programmed $D_{add}$ values.

**Fig. 1.**
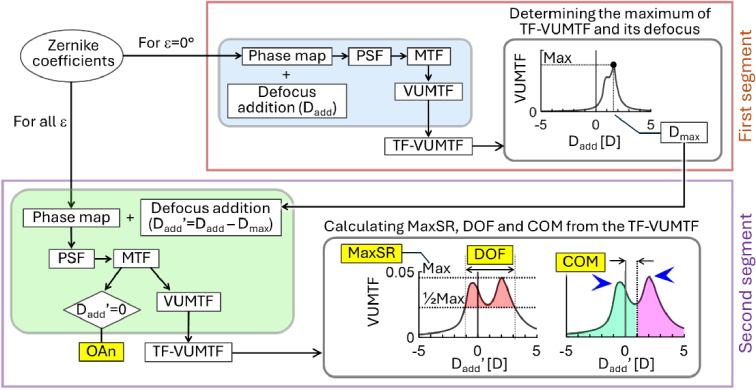
Data processing for calculating image-quality metrics. In the second segment, the blue arrows show the double peaks of VUMTF originating from the off-axis cylinder.

The calculation of the parameters characterizing the through-focus image quality (DOF, MaxSR, COM and OAn) from the Zernike coefficients for each subject, visit and eccentricity consisted of two sequential segments (see Fig. 1), as explained below.

In the first segment, the TF-VUMTF at the central retina (ε=0°) was calculated using the procedure described above and represented within the blue rectangle in Fig. 1 to identify the defocus value that maximizes the VUMTF. This value was set $D_{max}$ and will be used to calculate COM and OAn in the second part of the procedure, referring these parameters to the best focus at ε=0°. This operation mimics the effect of either accommodation in hyperopic eyes or corrected vision in myopic eyes.

In the second segment, DOF, MaxSR, COM and OAn were calculated for each ε. Initially, the TF-VUMTF was calculated from the Zernike coefficients for each ε using the procedure described above and shown within the green rectangle in Fig. 1. In this case, the defocus values of TF-VUMTFs (i.e., ${D_{add}}^{\prime }$ ) were referred to $D_{max}$ by subtracting $D_{max}$ to $D_{add}$ in Eq. (1). The TF-VUMTF was first processed to calculate its full width at half maximum and its overall maximum, corresponding to the DOF and MaxSR, respectively.

COM corresponded to the ${D_{add}}^{\prime }$ value at which the integrated VUMTF is balanced, such that the contributions over either side of this value are equal. COM was calculated as:

(4) $$\mathrm{COM}=\frac{\sum_{D_{\text{add }}=-5D}^{5D}{D_{\text{add }}}^{\prime }\mathrm{VUMTF}\left(D_{\text{add }}\prime \right)}{\sum_{\begin{array}{c} D_{\text{add }}=-5D\\ \text{ step }\Delta D_{\text{add }} \end{array}}^{5D}\mathrm{VUMTF}\left(D_{\text{add }}\prime \right)}$$

Positive and negative COM values correspond to the concentration of sharpest images in front of and behind the retina, respectively.

Lastly, the optical anisotropy at the retina (OAn) was calculated by:

(5) $$\text{OAn = }\frac{\sum_{j=1}^{N}\sum_{i=1}^{N}{MTF|}_{{D_{add}}^{\prime }=0}(i,j)circ(i,j)Mask_{H}(i,j)}{\sum_{j=1}^{N}\sum_{i=1}^{N}{MTF|}_{{D_{add}}^{\prime }=0}(i,j)circ(i,j)Mask_{V}(i,j)}$$

where $MTF{|}_{{D_{add}}^{\prime }=0}$ is the MTF matrix for ${D_{add}}^{\prime }$ =0, and $Mask_{H}$ and $Mask_{V}$ were binary functions that select the MTF values in horizontal and vertical directions. For each (i,j) position, $Mask_{V}$ and $Mask_{H}$ were set to 1 for |θ(i,j)|≤π/4 and |θ(i,j)|>π/4 , respectively, and to 0 otherwise, where θ(i,j) is the arctangent of (j−N/2)/(i−N/2) . Both $Mask_{V}$ and $Mask_{H}$ were valued zero for the (i,j) position corresponding to a zero spatial frequency. Logarithmic OA (Log_10_(OAn)) was used in our analysis, as it is more readily associated with the orientation of the PSF [17]. Positive, negative and zero values of Log_10_(OAn) correspond to vertical, horizontal or rotationally symmetric PSFs, respectively.

Section 1 of the Supplement 1 presents the relationships between DOF, MaxSR, COM, and OAn and established aberrometric metrics, including cylinder magnitude, the root mean square of higher-order aberrations, relative peripheral refraction, and spherical aberration.

### 2.2. Baseline and three-year changes in through-focus image quality

Baseline, final and changes in DOF, MaxSR, COM, and Log_10_(OAn) over the three-year study period were evaluated and related to changes in the cycloplegic central spherocylindrical error (cSE) and the ratio between the axial length to corneal radius ratio (AXL/CR), which was included due to its stronger correlation with cSE than AXL alone [18]. For this analysis, children were classified into three groups: LR, those with a low baseline risk of myopia who remained non-myopic; HR, those with a high baseline risk of myopia who remained non-myopic; and MYO, those who developed myopia (i.e., cSE < -0.50 D) or showed marked myopia-prone development during follow-up, quantified by a mean annual cSE change < -0.50 D/year, or a third-year cSE change < -0.75 D. Subjects with missing data on image quality, cSE, or AXL/CR at either the first or seventh visit were excluded.

The data analysis consists of the following tests:

A. Inter-group comparison (i.e., LR-HR, MYO-LR, and MYO-HR) of baseline, three-year changes and final cSE and AXL/CR values, as well as the ratio of baseline cSE to baseline age (cSE_bl_/age_bl_). cSE_bl_/age_bl_ serves as a simple metric to adjust for age-related effects on refractive error, enabling fairer comparisons of baseline refractive status. The data normality of each quantity was assessed using the Shapiro-Wilk test, while homogeneity of variances was evaluated using Levene’s test. Parametric (ANOVA or Welch’s t-test) or nonparametric (Mann-Whitney U test) tests were applied to evaluate the inter-group differences. To account for three inter-group comparisons per quantity, significance thresholds (or α values) were adjusted using the Holm-Bonferroni method.
B. For each through-focus image quality parameter and ε, inter-group differences in baseline values, three-year changes and final values of these parameters were evaluated. Statistical significances were assessed using the procedure described in A.
C. For each through-focus image quality parameter and ε, associations between baseline parameter values and 3-year changes in refractive descriptors (i.e., -ΔcSE and ΔAXL/CR) were evaluated within each group using Pearson correlation coefficients (PCCs) and corresponding p-values. To account for the three comparisons across the groups, α values were adjusted using the Holm-Bonferroni method.
D. Similarly to C, for each through-focus image quality parameter and ε, associations between 3-year changes in these parameters and refractive changes were evaluated within each group using PCCs and their corresponding p-values. To account for the three comparisons across the groups, α values were adjusted using the Holm-Bonferroni method.
E. For each through-focus image quality parameter and ε, intra-group baseline-to-final changes in these parameters were evaluated. Normality of the paired differences (final-baseline) was assessed using the Shapiro-Wilk test. Paired t-test or Wilcoxon signed-rank test were applied for normally or non-normally distributed data to calculate the p-values. α was set at 0.05 for each intra-group comparison.

## 3. Results

Of the 102 participants enrolled in the PICNIC study, 92 completed at least three biannual visits over the three-year study duration. For the analysis presented in this manuscript, participants were excluded if cSE, AXL, CR, or through-focus image quality data across the full eccentricity range were missing at the first or seventh (i.e., final) visit. Based on these criteria, 20 of 48 participants in the LR group and 10 of 30 in the HR group were excluded; no MYO participants were excluded. In total, 62 children were included in this analysis. Table 1 summarizes the characteristics of the three eye groups (LR, HR, and MYO), including the sample size. The HR subjects were significantly younger than LR subjects (p = 0.006, α =0.017, |z|=2.77 for LR-HR; p = 0.11, α =0.050, |t|=1.65 for HR-MYO; p = 0.15, α =0.025, |z|=1.44 for LR-MYO). The baseline cSE values differed significantly among the groups (p < 0.001, α ≥0.017; |t|=3.67 for LR-HR; |z|=3.62 for HR-MYO; |z|=4.75 for LR-MYO). Likewise, the final cSE values were significantly different among the groups (p < 0.001, α ≥0.017; |t|=3.89 for LR-HR; |t|=9.30 for HR-MYO; |t|=12.72 for LR-MYO). On the other hand, the baseline AXL/CR values did not differ significantly among the groups (p = 0.714, α =0.050, |z|=0.37 for LR-HR; p = 0.245, α =0.025, |t|=1.19 for HR-MYO; p = 0.044, α =0.017, |z|=2.01 for LR-MYO). The final AXL/CR in the MYO group was significantly higher than LR (p < 0.001, α =0.017, |t|=4.36) and HR (p = 0.013, α =0.025, |t|=2.62) groups, but no significant difference was found between the LR and HR groups (p = 0.291, α =0.050, |t|=1.07).

**Table 1.** Characteristics of the study groups: children with a low baseline risk of myopia who remained non-myopic (LR), children with a high baseline risk of myopia who remained non-myopic (HR), and children who developed myopia or showed marked myopic progression during follow-up (MYO). Age, cSE and AXL/CR values are reported as mean ± standard deviation. Race was classified as Asian (A), Black (B), White (W), or multiracial (MR).

		LR	HR	MYO
Sample size		28	20	14
Baseline age [years]		7.7 ± 1.0	6.9 ± 0.8	7.4 ± 0.9
Sex		Female (50%)	Female (60%)	Female (64%)
		Male (50%)	Male (40%)	Male (36%)
Race		A(0%), B(14%), W(68%), MR(18%)	A(15%), B(10%), W(30%), MR(45%)	A(21%), B(21%), W(37%), MR(21%)
cSE [D]	Baseline	1.36 ± 0.46	0.90 ± 0.39	0.29 ± 0.37
	Final	1.14 ± 0.39	0.72 ± 0.35	-0.65 ± 0.51
AXL/CR	Baseline	2.94 ± 0.06	2.94 ± 0.07	2.97 ± 0.06
	Final	2.97 ± 0.05	3.00 ± 0.07	3.06 ± 0.07

**Fig. 2.**
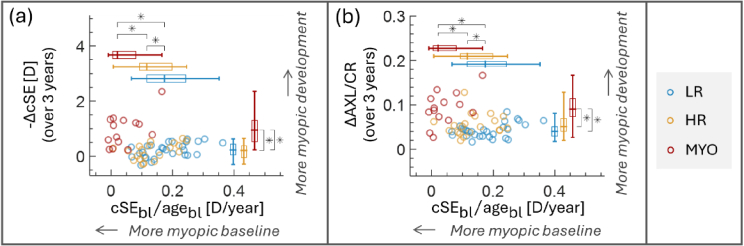
Baseline and three-year changes in the parameters of through-focus image quality in the low risk (LR), high risk (HR) and myopia (MYO) groups. Three-year changes of (a) the negative central spherical error (-ΔcSE) and (b) the ratio between the axial length and the corneal radius (ΔAXL/CR), as a function of the baseline ratio between cSE and age (cSE_bl_/age_bl_). * indicates significant differences.

Figure 2 shows the three-year changes in -cSE (-ΔcSE) and AXL/CR (ΔAXL/CR) as a function of the ratio of baseline cSE to baseline age (cSE_bl_/age_bl_). The significant differences of cSE_bl_/age_bl_ among the groups (p = 0.03, α =0.05, |t|=2.25 for LR-HR; p < 0.001, α ≥0.017, |z|=3.52 for HR-MYO; p < 0.001, α =0.017, |z|=4.52 for LR-MYO) reflect different refractive development patterns at baseline. After 3 years, refractive developments led ΔAXL/CR and -ΔcSE values significantly higher in MYO than LR (for ΔAXL/CR, p < 0.001, α =0.017, |t|=4.85; and for -ΔcSE, p < 0.001, α =0.025, |t|=4.40) and HR (for ΔAXL/CR, p = 0.003, α =0.025, |z|=3.00; and for -ΔcSE, p < 0.001, α =0.017, |t|=4.52) groups. ΔAXL/CR and -ΔcSE were not significantly different between the LR and HR groups (for -ΔcSE, p = 0.61, α =0.05, |t|=0.52; for ΔAXL/CR, p = 0.05, α =0.05, |z|=2.00).

### 3.1. Baseline and three-year changes in DOF

**Fig. 3.**
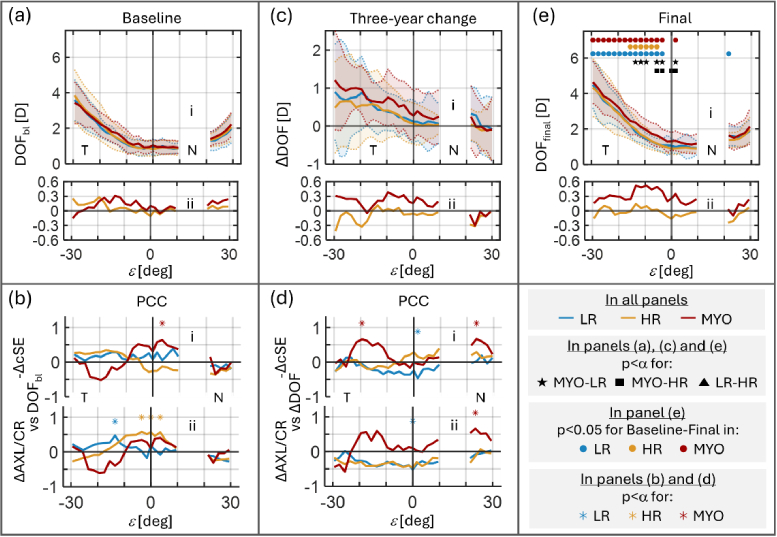
Baseline and three-year changes of depth-of-focus (DOF) in the low myopia-risk (LR), high myopia-risk (HR) and myopia (MYO) groups, as a function of the eccentricity ε. (a.i) Mean (solid line) and standard deviation (shadow) of the baseline DOF (DOF_bl_) in the three groups. (a.ii) Difference of the mean DOF_bl_ in MYO and HR with respect to LR. (b) Pearson correlation coefficients (PCC) of the linear regressions between: (b.i) -ΔcSE and DOF_bl_, and (b.ii) ΔAXL/CR and DOF_bl_. (c.i) Mean (solid line) and standard deviation (shadow) of the three-year change of DOF (ΔDOF) for the three groups. (c.ii) Difference of the mean ΔDOF in MYO and HR with respect to LR. (d) PCC of the linear regressions between: (d.i) -ΔcSE and ΔDOF, and (d.ii) ΔAXL/CR and ΔDOF. (e.i) Mean (solid line) and standard deviation (shadow) of the final values of DOF (DOF_final_) for the three groups. (e.ii) Difference of the mean DOF_final_ in MYO and HR with respect to LR. Statistics for inter-group comparisons in (a.i), (b.i), (b.ii), (c.i), (d.i), (d.ii) and (e.i) are reported in Tables S2, S18, S19, S3, S26, S27 and S4 of Supplement 1, respectively. Statistics for intra-group comparisons of DOF_final_-DOF_bl_ in (e.i) are reported in Table S14 of Supplement 1. T and N denote temporal and nasal retinal eccentricities, respectively.

Figure 3(a) shows baseline DOF (DOF_bl_) as a function of ε. In general, DOF was asymmetric along the horizontal meridian, with higher values at the temporal retina than at the nasal retina across all groups. DOF_bl_ showed no significant differences among the groups. Figure 3(b) shows that in MYO, greater myopic development (i.e., higher -ΔcSE and ΔAXL/CR) was associated with longer and shorter DOF_bl_ at the central and temporal retina, respectively; however, these associations reached statistical significance only at ε=3.5°.

Figure 3(c) shows the three-year changes of DOF (ΔDOF) as a function of ε. In all groups, ΔDOF showed a trend towards higher in the MYO than HR and LR groups in the temporal retina, with no significant differences among groups. Slope and PCCs from linear regressions of ΔDOF with -ΔcSE and ΔAXL/CR were calculated using intra-group data, showing PCCs in Fig. 3(d). These regressions indicate that higher -ΔcSE (i.e., greater myopic development) in MYO was associated with increases in temporal (ε∼-19.5°) and nasal (ε∼23.5°) DOF, with mean rates of 1 D in ΔDOF per 0.5 D in -ΔcSE. On the other hand, LR eyes with higher ΔAXL/CR (i.e., less reduction in hyperopia) exhibited a decrease of the central DOF with a mean rate of -1 D in ΔDOF per 0.015 units of ΔAXL/CR.

Lastly, the mean final DOFs (DOF_final_) are shown in Fig. 3(e). The DOF_final_-DOF_bl_ difference was highly significant in the temporal retina in all groups.

### 3.2. Baseline and three-year changes in MaxSR

**Fig. 4.**
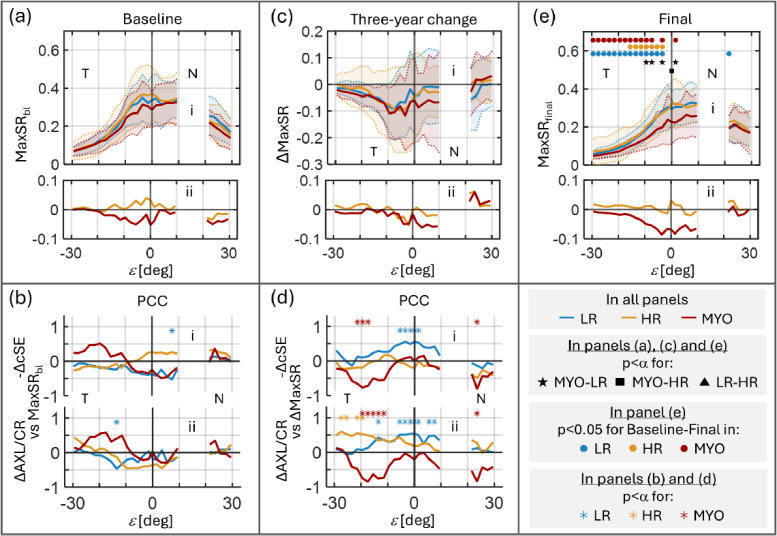
Baseline and three-year changes of the maximum through-focus Strehl ratio (MaxSR) in the low myopia-risk (LR), high myopia-risk (HR) and myopia (MYO) groups, as a function of the eccentricity ε. (a.i) Mean (solid line) and standard deviation (shadow) of the baseline MaxSR (MaxSR_bl_) for the three groups. (a.ii) Difference of the mean MaxSR_bl_ in MYO and HR with respect to LR. (b) Pearson correlation coefficients (PCC) of the linear regressions between: (b.i) -ΔcSE and MaxSR_bl_, and (b.ii) ΔAXL/CR and MaxSR_bl_. (c.i) Mean (solid line) and standard deviation (shadow) of the three-year change of MaxSR (ΔMaxSR) for the three groups. (c.ii) Difference of the mean ΔMaxSR in MYO and HR with respect to LR. (d) PCC of the linear regressions between: (d.i) -ΔcSE and ΔMaxSR, and (d.ii) ΔAXL/CR and ΔMaxSR. (e.i) Mean (solid line) and standard deviation (shadow) of the final values of MaxSR (MaxSR_final_) for the three groups. (e.ii) Difference of the mean MaxSR_final_ in MYO and HR with respect to LR. Statistics for inter-group comparisons in (a.i), (b.i), (b.ii), (c.i), (d.i), (d.ii) and (e.i) are reported in Tables S5, S20, S21, S6, S28, S29 and S7 of Supplement 1, respectively. Statistics for intra-group comparisons of MaxSR_final_-MaxSR_bl_ in (e.i) are reported in Table S15 of Supplement 1. T and N denote temporal and nasal retinal eccentricities, respectively.

Figure 4(a) shows baseline MaxSR (MaxSR_bl_) as a function of ε. Mean MaxSR_bl_ appeared lower in MYO than in LR and HR, but the differences were not statistically significant. PCCs from the linear regressions of MaxSR_bl_ with -ΔcSE and ΔAXL/CR (see Fig. 4(b)) closely correspond to the inverse of those values shown in Fig. 3(b), given the inverse relationship between MaxSR and DOF (see Section 1 of Supplement 1). In LR eyes, the associations between refractive descriptors and MaxSR_bl_ were not consistently localized. Specifically, statistical significance between -ΔcSE and MaxSR_bl_ wass reached at ε = 7.5°, whereas significance between ΔAXL/CR and MaxSR_bl_ was observed at ε = -13.5.

The three-year changes of MaxSR (ΔMaxSR) are shown in Fig. 4(c). The mean ΔMaxSR was lower at ε ≈ -7.5° for all groups, suggesting an accentuated degradation of image quality at this eccentricity, even under normal refractive development. ΔMaxSR was not significantly different among the groups. Figure 4(d) shows the PCCs from the linear regressions between ΔMaxSR with -ΔcSE and ΔAXL/CR. These relationships showed that higher MaxSR degradations at the temporal and nasal retina characterized MYO eyes with greater myopic development. Conversely, LR eyes with higher ΔAXL/CR and -ΔcSE (i.e., more myopic development) were associated with increased MaxSR in the central retina. In HR eyes, ΔMaxSR showed a significant association with ΔAXL/CR at the temporal retina; however, this relationship was not consistently observed with -ΔcSE.

These changes led to a mean three-year MaxSR (MaxSR_final_) that was lower in MYO than in HR and LR, with significant differences in the central retina (see Fig. 4(e)). Similar to DOF in Fig. 3(e), MaxSR_final_-MaxSR_bl_ differences were significant in the temporal retina of all groups.

### 3.3. Baseline and three-year changes in COM

**Fig. 5.**
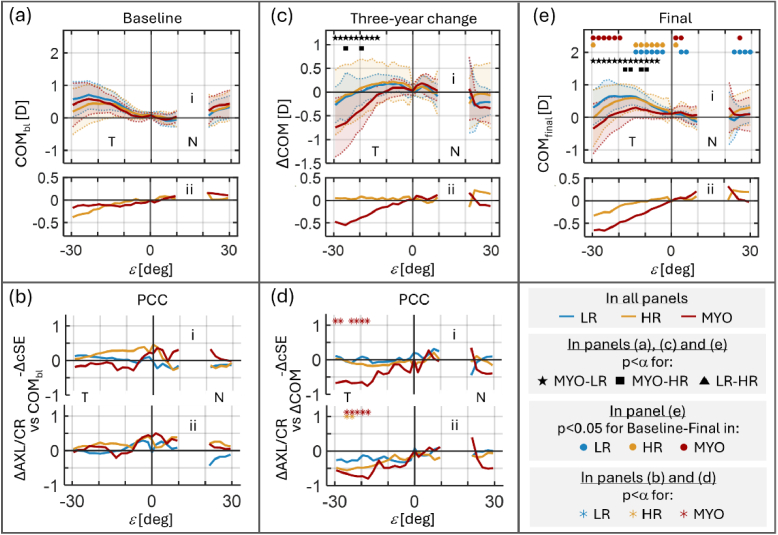
Baseline and three-year changes of the center of mass (COM) in the low myopia-risk (LR), high myopia-risk (HR) and myopia (MYO) groups, as a function of the eccentricity ε. (a.i) Mean (solid line) and standard deviation (shadow) of the baseline COM (COM_bl_) for the three groups. (a.ii) Difference of the mean COM_bl_ in MYO and HR with respect to LR. (b) Pearson correlation coefficients (PCC) of the linear regressions between: (b.i) -ΔcSE and COM_bl_, and (b.ii) ΔAXL/CR and COM_bl_. (c.i) Mean (solid line) and standard deviation (shadow) of the three-year change of COM (ΔCOM) for the three groups. (c.ii) Difference of the mean ΔCOM in MYO and HR with respect to LR. (d) PCC of the linear regressions between: (d.i) -ΔcSE and ΔCOM, and (d.ii) ΔAXL/CR and ΔCOM. (e.i) Mean (solid line) and standard deviation (shadow) of the final values of COM (COM_final_) for the three groups. (e.ii) Difference of the mean COM_final_ in MYO and HR with respect to LR. Statistics for inter-group comparisons in (a.i), (b.i), (b.ii), (c.i), (d.i), (d.ii) and (e.i) are reported in Tables S8, S22, S23, S9, S30, S31 and S10 of Supplement 1, respectively. Statistics for intra-group comparisons of COM_final_-COM_bl_ in (e.i) are reported in Table S16 of Supplement 1. T and N denote temporal and nasal retinal eccentricities, respectively.

Figure 5(a) shows baseline COM (COM_bl_) as a function of ε. Mean COM_bl_ across all groups exhibited positive (i.e., myopic) values at the temporal (peak at ε=-23.5°) and nasal (at ε>21.5°) retina, without significant differences among the groups. In addition, Fig. 5(b) shows no significant associations between either -ΔcSE or ΔAXL/CR and COM_bl_.

Figure 5(c) shows the three-year changes of COM (ΔCOM). ΔCOM was significantly hyperopic in MYO compared to LR and HR at the temporal retina, while those differences were null at the nasal retina. Figure 5(d) shows the PCCs from the linear regressions between ΔCOM with -ΔcSE and ΔAXL/CR. According to these regressions, ΔCOM significantly decreases at the temporal retina in MYO eyes with more myopic development, with mean rates of ∼0.9 D in ΔCOM per 1 D in -cΔSE or ∼0.07 units of ΔAXL/CR. In HR eyes, higher ΔAXL/CR was significantly associated with a hyperopic shift of COM at ε around -24.5°; however, this relationship was not consistently observed with -ΔcSE.

After 3 years, the mean final COM values (COM_final_) in MYO become flatter than in the LR and HR groups, as seen in Fig. 5(e). This final profile indicates that the peripheral blur in accommodated or corrected myopic eyes will be lower than in emmetropic eyes. The significance of the COM_final_-COM_bl_ difference varies by group: it was observed at near-temporal and nasal eccentricities in LR, near-temporal eccentricities in HR, and far-temporal eccentricities in MYO.

### 3.4. Baseline and three-year changes in OAn

**Fig. 6.**
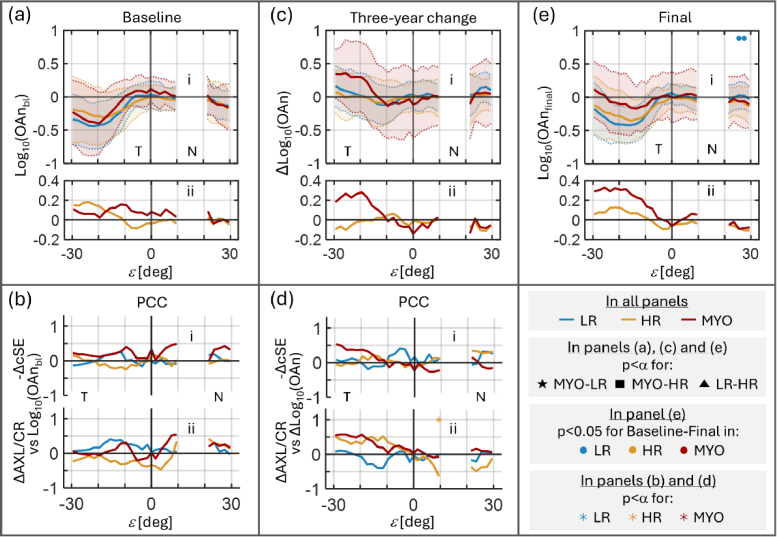
Baseline and three-year changes of the logarithmic optical anisotropy (Log_10_(OAn)) in the low myopia-risk (LR), high myopia-risk (HR) and myopia (MYO) groups, as a function of the eccentricity ε. (a.i) Mean (solid line) and standard deviation (shadow) of the baseline Log_10_(OAn) (Log_10_(OAn_bl_)) for the three groups. (a.ii) Difference of the mean Log_10_(OAn_bl_) in MYO and HR with respect to LR. (b) Pearson correlation coefficients (PCC) of the linear regressions between: (b.i) -ΔcSE and Log_10_(OAn_bl_), and (b.ii) ΔAXL/CR and Log_10_(OAn_bl_). (c.i) Mean (solid line) and standard deviation (shadow) of the three-year change of Log_10_(OAn) (ΔLog_10_(OAn)) for the three groups. (c.ii) Difference of the mean ΔLog_10_(OAn) in MYO and HR with respect to LR. (d) PCC of the linear regressions between: (d.i) -ΔcSE and ΔLog_10_(OAn), and (d.ii) ΔAXL/CR and ΔLog_10_(OAn). (e.i) Mean (solid line) and standard deviation (shadow) of the final values of Log_10_(OAn) (Log_10_(OAn_final_)) for the three groups. (e.ii) Difference of the mean Log_10_(OAn_final_) in MYO and HR with respect to LR. Statistics for inter-group comparisons in (a.i), (b.i), (b.ii), (c.i), (d.i), (d.ii) and (e.i) are reported in Tables S11, S24, S25, S12, S32, S33 and S13 of Supplement 1, respectively. Statistics for intra-group comparisons of Log_10_(OAn_final_)-Log_10_(OAn_bl_) in (e.i) are reported in Table S17 of Supplement 1. T and N denote temporal and nasal retinal eccentricities, respectively.

Figure 6(a) shows baseline Log_10_(OAn) (Log_10_(OAn_bl_)) as a function of ε. All groups exhibited negative mean Log_10_(OAn_bl_) values in the peripheral retina, without significant differences among them. Log_10_(OAn_bl_) was not statistically associated with ΔAXL/CR and ΔcSE, as indicated by Fig. 6(b).

Figure 6(c) shows three-year changes of Log_10_(OAn) (ΔLog_10_(OAn)). Mean ΔLog_10_(OAn) at the temporal retina was higher in MYO than LR and HR, but without statistical significance. The linear regression analyses of ΔLog_10_(OAn) versus -ΔcSE and ΔAXL/CR, shown in Fig. 6(d), revealed that MYO eyes with greater myopic developed were associated with an increase in OAn, but without statistical significance. In HR eyes, ΔAXL/CR was significantly associated with ΔLog_10_(OAn) at ε=9.5°; however, this relationship was not consistently observed with -ΔcSE.

Mean final Log_10_(OAn) values (Log_10_(OAn_final_)) are shown in Fig. 6(e). Log_10_(OAn_final_) at the temporal retina were higher in MYO than LR and HR, leading to a more uniform distribution of blur orientation over the retina, but without statistical significance. Only Log_10_(OAn_final_) was significantly different from Log_10_(OAn_bl_) in LR at ε=27.5°.

## 4. Discussion

To our knowledge , this is the first study to characterize both baseline and longitudinal changes in through-focus image quality across a continuous broad range of eccentricities in school-aged children with baseline functional emmetropia who later diverged into distinct refractive development trajectories, and to assess their associations with key refractive descriptors.

Three main findings that characterize myopia development stand out. First, DOF increased significantly, whereas MaxSR decreased significantly in the peripheral retina, even after correction for foveal defocus. Second, TF-VUMTF in the temporal retina exhibited a hyperopic shift, with a mean change of 0.5 D per -1.0 D in ΔcSE. Third, the optical anisotropy at the retinal image plane (OAn) showed a statistically non-significantly increase, again more so in the temporal retina, which leads to a more uniform distribution of blur orientation across the retina. These and other findings are discussed below in relation to previous experimental and clinical studies.

Classic emmetropization theory proposes that the retina uses defocus-related signals to regulate axial growth [12,19]. Consequently, most animal and human studies of myopia have focused on relative peripheral refraction and assumed simple on-retina blur profiles. Our findings extend this framework by showing that refractive development is associated not only with the sign of peripheral defocus, but also with systematic changes in the distribution of usable image quality across dioptric space – captured by DOF, MaxSR and COM. Two possible hypotheses emerge from these results:

• *Signal dilution hypothesis.* Ocular aberrations, including cylinder, flatten the TF-VUMTF and decrease its peak amplitude (i.e., increasing DOF while decreasing MaxSR). During myopic development, these aberrations lead to greater flattening, producing a more uniform and symmetric blur distribution in the axial regions immediately anterior and posterior to the retina. Such an effect may reduce sensitivity to defocus and degrade the fidelity of retinal signals that regulate eye growth [12]. In simple terms, increasing DOF would lead to myopic refractive development. Findings from animal [12] and human [20] experiments support this hypothesis. First, the retina can detect the sign of defocus by exploiting asymmetries in the through-focus retinal blur caused by ocular aberrations [12]. Second, increasing DOF when projecting either hyperopic or myopic retinal stimuli induces short-term choroidal thinning [20], which is considered an early biomarker of eye elongation or myopia development [21]. Our findings also partially support this hypothesis. Specifically, Figs. 3(d) and 4(d) show that greater myopic progression in MYO eyes was associated with increases in DOF and decreases in MaxSR at specific retinal eccentricities (ε=-19.5° and 23.5°), consistent with this hypothesis. In contrast, Figs. 3(b) and 4(b) show that narrower baseline DOF and higher baseline MaxSR at ε∼-19.5° were associated, albeit non-significantly, with greater myopic development in MYO eyes. This trend appears inconsistent with this hypothesis, as these eyes already exhibited more myopic central refraction at baseline (Fig. 2). A recent study reported no significant differences in DOF at ε=±25° between myopes and non-myopes in 19 adults (including 9 myopes) and 33 children (including only 5 myopes) [22], seemingly weakening this hypothesis. However, our results suggest that this lack of significance may be attributable to variability in the associations of -ΔcSE and ΔAXL/CR with changes in DOF and MaxSR across ε. For example, although the correlations of -ΔcSE with ΔDOF (Fig. 3(d).i) and ΔMaxSR (Fig. 4(d).i) were strong and significant at ε=-19.5°, they were not significant at ε=-25.5°. Moreover, our inter-group comparison of 3-year far-peripheral DOF (see Fig. 3(e)) was also not significant. Taken together, these findings indicate that further longitudinal studies investigating central and peripheral DOF and MaxSR in larger cohorts are needed to test this hypothesis conclusively.
• *Directional bias hypothesis.* The center of mass (COM) of the TF-VUMTF accounts for which side of the retina (in front of or behind) accumulates the sharpest images. In this work, negative or hyperopic COM values imply that the image is focused behind the retina relative to the foveal focus. Figure 5(b) showed that central refractive changes were not associated with baseline COM values. This finding aligns with previous studies concluding that myopia onset cannot be predicted from baseline relative peripheral refraction (RPR) [23–26], which is highly correlated with COM (see Figure S2(d) in Supplement 1). The significant correlations shown in Fig. 5(d) indicate that the hyperopic shift of COM at temporal eccentricities was a consequence of the myopia development/progression.

Our findings and hypotheses do not contradict the clinical outcomes showing that extended-DOF contact lenses slow myopia progression (e.g., [27–29]). These lenses use discrete or continuous dioptric-power profiles to induce myopic shifts in COM at the central or peripheral retina [30] – rather than relying solely on DOF extension – to reduce myopia progression, consistent with the simultaneous competing blur theory [13].

Furthermore, our findings may help clarify the mechanism underlying the therapeutic mechanisms of orthokeratology (Ortho-K) on myopia progression. Although it has been suggested that Ortho-K slows myopia by extending the peripheral DOF, based on reports of increased peripheral HO-RMS in Ortho-K wearers compared with spectacles wearers [11,31], our interpretation suggests that a myopic shift of peripheral COM could be the primary mechanism rather than a DOF extension. Two observations support this hypothesis. First, Ortho-K induces a myopic shift in peripheral refractive error through corneal reshaping [11], thereby shifting the peripheral COM in the myopic direction. Second, peripheral DOF is mainly driven by peripheral cylinder rather than HO-RMS (see Figure S2(b) in Supplement 1). Because Ortho-K and spectacle wearers showed similar levels of peripheral cylinder [11], we expect any DOF differences between groups to be small. In summary, several optical myopia-control interventions are often described as extending DOF, but their primary optical effect appears to be a modification of COM.

This work underscores the value of measuring central and peripheral ocular aberrations in children for monitoring their refractive development and informing the enhancement of optical interventions for myopia control. Monitoring sensitivity may be improved by incorporating location-specific changes in through-focus image quality metrics derived from aberrations, particularly when conventional measures such as RPR or AXL velocity are inconclusive. Notable early changes include: (i) a reduction of MaxSR at the central and near-temporal retina, consistent with findings that myopes with optimal foveal refractive correction exhibit lower MTF values than emmetropes in temporal regions [28]; and (ii) a hyperopic shift in COM and increased OAn at the temporal retina, in line with reports of relative peripheral hypermetropia in older myopic eyes [17,32,33]. Conversely, our results suggest that refractive development is not robustly predictable from baseline data of each image quality parameter. Baseline COM and Log_10_(OAn) were not significantly associated with either ΔcSE or ΔAXL/CR (see Figs. 5(b) and 6(b)). Moreover, although consistent associations were observed between baseline DOF and MaxSR and the quantified changes in refractive development in MYO eyes (Figs. 3(b) and 4(b)), these associations were generally not statistically significant and, as discussed above, were inconsistent with the signal dilution hypothesis. Consequently, these findings weaken the potential utility of DOF and MaxSR as standalone predictive markers.

The main study limitation is the small sample size for children who developed myopia during the study duration. Therefore, the replication of this analysis in larger cohorts is required. In addition, the central and peripheral aberrations analyzed in this work were measured while the children fixated on a distant target (6 m) to minimize accommodative fluctuations. However, some residual accommodation variability may have persisted, potentially contributing to the observed variability in through-focus image quality parameters.

Future work will aim to extend this longitudinal analysis to additional through-focus image quality parameters. One promising metric is depth-of-refraction, recently introduced to quantify the range of refraction over which image quality remains preserved when sphero-cylindrical wavefronts are added; this metric has shown greater sensitivity than DOF in distinguishing myopic from non-myopic children [22]. Moreover, investigating peripheral multifocality [22] in our dataset may provide further insights into the simultaneous competing blur theory in children. Another avenue is the development of polychromatic, task-relevant models that integrate through-focus optics with neural weighting, such as pediatric contrast sensitivity functions, to estimate visual Strehl through focus and potentially improve predictive performance for refractive development. It will also be important to relate our optical findings to regional eye-shape remodeling using OCT-based posterior pole morphometry, which is available for these children.

In conclusion, children who progress toward myopia exhibit distinct, eccentricity-specific changes in through-focus image quality both before and during onset: peripheral DOF widens, central MaxSR decreases and temporal COM shifts hyperopically. Together, these features offer a coherent optical narrative in which the retina experiences a broader and directionally biased range of “acceptable” focus, potentially weakening emmetropization cues. Conceptualizing myopia control in terms of how ocular optics shape the through-focus landscape may enhance risk prediction and guide the development of future personalized optical interventions.

## Supplemental information

Supplement 1 Supplement https://doi.org/10.6084/m9.figshare.32415810

## Data Availability

Data underlying the results presented in this paper are not publicly available at this time but may be obtained from the authors upon reasonable request.

## Supplemental document

See Supplement 1 for supporting content.
