# Supplementary material for: Longitudinal changes in retinal image quality in school-aged children
Source: Biomed Opt Express. 2026 Jul 2;17(8):4036–49. doi: 10.1364/BOE.587977 (PMC13481062; doi:10.1364/BOE.587977)
Supplement: Supplementary file 1 [file boe-17-8-4036-s001.pdf]

Supplemental Document

Biomedical Optics EXPRESS

Longitudinal changes in retinal image quality in school-aged children: supplement

AUGUSTO ARIAS,[1,*] SUSANNA P. CLEMENT,[1] SIEGFRIED WAHL,[1,2] AND FUENSANTA A. VERA-DIAZ[3]

[1] *ZEISS Vision Science Lab, University of Tübingen, Tübingen, Germany*
[2] *Carl Zeiss Vision International GmbH, Aalen, Germany*
[3] *Children's Vision Lab, New England College of Optometry, Boston, MA, USA*
[*] *augusto.arias-gallego@uni-tuebingen.de*

This supplement published with Optica Publishing Group on 2 July 2026 by The Authors under the terms of the Creative Commons Attribution 4.0 License in the format provided by the authors and unedited. Further distribution of this work must maintain attribution to the author(s) and the published article's title, journal citation, and DOI.

Supplement DOI: https://doi.org/10.6084/m9.figshare.32415810

Parent Article DOI: https://doi.org/10.1364/BOE.587977

LONGITUDINAL CHANGES IN RETINAL IMAGE QUALITY IN SCHOOL-AGED CHILDREN

This Supplemental Material is organized as follows:

- Section 1 shows the associations between the through-focus image quality parameters and commonly known Zernike-coefficients-based metrics.
- Section 2 shows tables containing the summary of the statistics used to evaluate the significance of differences in each through-focus retinal image quality parameter, at each eccentricity ε, among the three eye groups: those with an initial low risk of myopia but who did not develop it (LR), those with an initial high risk of myopia but who did not develop it (HR), and those who became myopic by the end of the study (MYO).

1. Optical factors influencing the through-focus image quality parameters

Associations between the through-focus image quality parameters and metrics directly derived from the Zernike coefficients were assessed using Spearman's rank correlation coefficients (SRCCs) and corresponding p-values across data from the seven visits of 92 subjects who participated in the PICNIC Study [1,2]. The through-focus image quality parameters were: depth-of-focus (DOF), maximum of the Strehl ratio (MaxSR), center of mass (COM), and the logarithm of the optical anisotropy at the retinal plane (Log_{10}(OAn)). The Zernike-coefficients-based metrics were: the cylinder magnitude (Cyl), the root-mean-square of high-order aberrations (HO-RMS), the relative peripheral refraction (RPR), and the spherical aberration (SA). Table S1 lists the formulae of these metrics. Initially, SRCCs between DOF, MaxSR, COM and Log_{10}(OAn) with Cyl, HO-RMS, RPR and SA were computed calculated using data from all 26 values of ε, except at ε=0º for RPR. To account for the comparisons for each image quality parameter, significance thresholds (α) were adjusted using the Holm-Bonferroni method. Subsequently, SRCCs were evaluated separately for each ε. In this stage, α was calculated based on the number of metrics tested for each image-quality parameter.

Table S1. Zernike-coefficients-based metrics correlated with the parameters of through-focus image quality. $C_k^m(\varepsilon)$, Zernike coefficients, at each eccentricity ε, with radial and azimuthal orders k and m respectively. $\emptyset$, pupil size.

Optical factor	Symbol	Calculation
Cylinder magnitude	$\mathrm{Cyl}(\varepsilon)$	$-\frac{16\sqrt{6}}{\emptyset^2}\sqrt{\left(C_2^{+2}(\varepsilon)\right)^2+\left(C_2^{-2}(\varepsilon)\right)^2}$
Root-mean-square of high-order aberrations	$\mathrm{HO\text{-}RMS}(\varepsilon)$	$\sqrt{\sum_{k=3}^{5}\sum_{m=-k}^{k}\left(C_k^m(\varepsilon)\right)^2}$
Relative peripheral refraction	$\mathrm{RPR}(\varepsilon)$	$-\frac{16\sqrt{3}}{\emptyset^2}\left(C_2^0(\varepsilon)-C_2^0(\varepsilon=0°)\right)$
Spherical aberration	$\mathrm{SA}(\varepsilon)$	$C_4^0(\varepsilon)$

The quantified associations are shown in Figure S1. Figure S1a shows SRCCs between the through-focus image quality parameters (DOF, MaxSR, COM and Log_{10}(OAn)) and the Zernike-coefficients-based metrics (HO-RMS, RPR, Cyl and SA), across all ε, except at ε=0º for RPR. DOF and MaxSR were strongly correlated with Cyl and weakly correlated with HO-RMS; both relationships were statistically significant (p<0.001, α=0.0125) but exhibited opposite trends. This divergence indicates a strong inverse relationship between DOF and MaxSR, as confirmed by a separate post hoc analysis (SRCC= −0.96, p < 0.001), showing that

broadening the TF-VUMTF is associated with a reduction in its peak amplitude. COM and Log_{10}(OAn) were primarily associated with RPR ($p<0.001$). The inverse dependence between COM and RPR means that a more hyperopic RPR results in a more myopic shift in COM.

Figures S1b-e show the SRCCs between the through-focus image quality parameters and the Zernike-coefficients-based metrics, as a function of ε. Cyl consistently determined DOF across the entire ε range, although the central cylinder Cyl(ε=0º) alone does not account for the peripheral DOF (i.e., for $|\varepsilon|>5°$), as seen in Figure S1b. HO-RMS had little effect on DOF in the central retina. The dependencies of MaxSR resemble the SRCC profiles of DOF, but with opposite sign (see Figure S1c). On the other hand, while COM was correlated with SA in the central retina, COM became strongly dependent on the RPR in the peripheral retina, as shown in Figure S1d. The positive correlation found between COM and SA means that more positive SAs myopically shift COM. Log_{10}(OAn) exhibited an asymmetric dependence on RPR, with SRCC>0.5 for ε<-5.5° in the temporal retina and for ε>~23.5° in the nasal retina. The correlation between Log_{10}(OAn) and Cyl was low across ε.

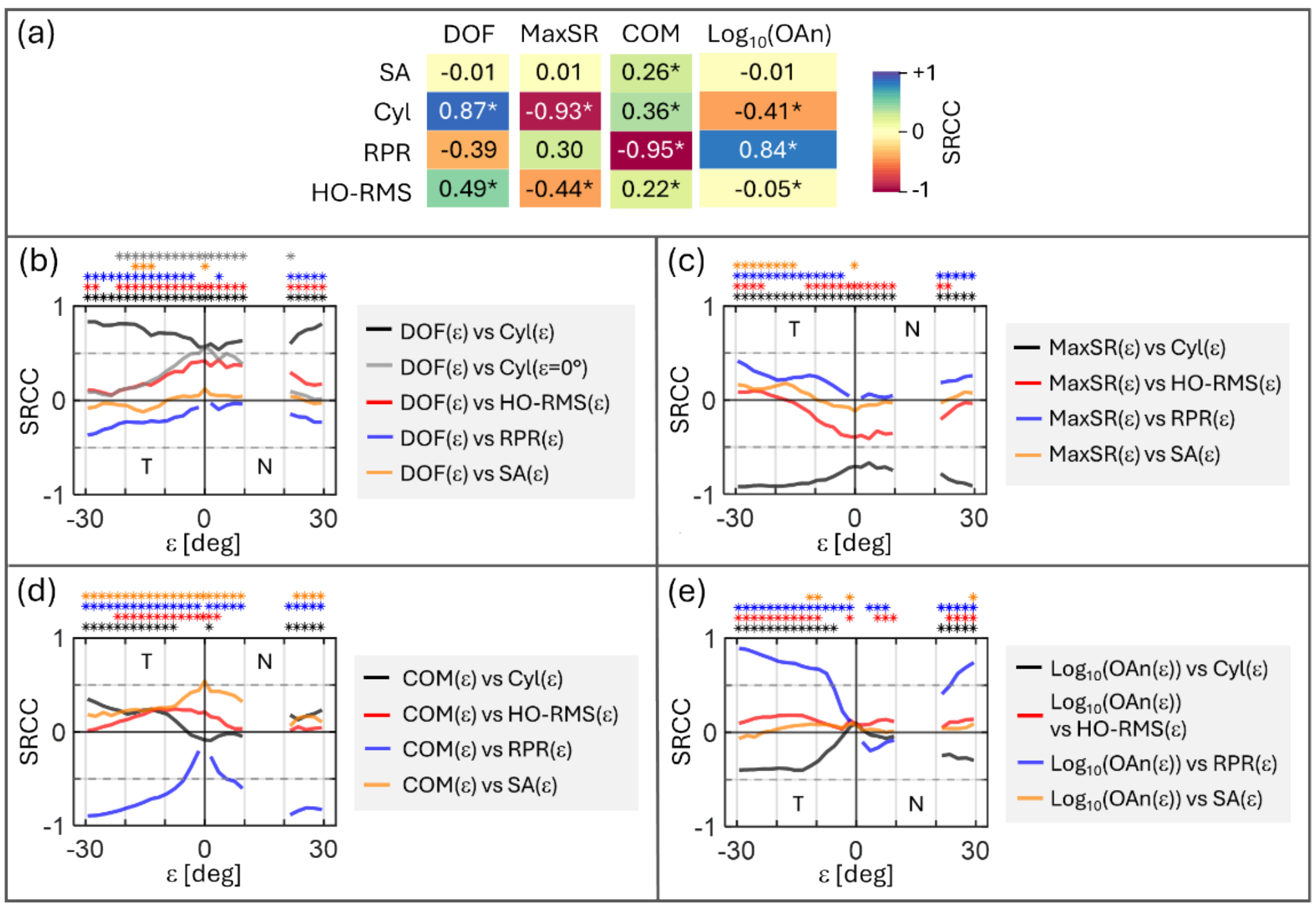

Figure S1. Dependency of through-focus image quality parameters (DOF, MaxSR, COM and Log_{10}(OAn)) on the root-mean-square of high-order aberrations (HO-RMS), relative peripheral refraction (RPR), cylinder (Cyl) and spherical aberration (SA). (a) Spearman's rank correlation coefficient (SRCC) of these dependencies across all eccentricities. (b), (c), (d) and (e) respectively show the SRCC of DOF, MaxSR, COM and Log_{10}(OAn) with the Zernike-coefficients-based metrics, as a function of the eccentricity ε. * indicates significant dependencies. T and N in (b)-(e) denote the temporal and nasal retinal eccentricities, respectively.

2. Summary of the statistics in the longitudinal analysis of the through-focus retinal image quality parameters and their associations with the refractive development

2.1 Inter-group differences in baseline, 3-year change and final through-focus image quality parameters

Table S2. Statistics used to assess the inter-group differences of baseline depth-of-focus (DOF_{bl}), at each eccentricity (ε). p, probability value; α, significance threshold adjusted using the Holm-Bonferroni method; t, t-statistic for paired t-tests; df, degrees of freedom associated with each t-test; and z, standardized statistic for Wilcoxon tests. * denotes p<α.

ε [deg]	LR vs. HR				MYO vs. LR				MYO vs. HR			
	p	α	|t| (df)	|z|	p	α	|t| (df)	|z|	p	α	|t| (df)	|z|
-29.5	0.496	0.025	0.69 (46)		0.690	0.050	0.4 (40)		0.390	0.017	0.87 (32)	
-27.5	0.380	0.017		0.88	0.612	0.025		0.51	0.737	0.050	0.34 (32)	
-25.5	0.565	0.025		0.58	0.557	0.017		0.59	0.779	0.050	0.28 (32)	
-23.5	0.587	0.017		0.54	0.650	0.025		0.45	0.814	0.050	0.24 (32)	
-21.5	0.587	0.017		0.54	0.603	0.025		0.52	0.698	0.050	0.39 (32)	
-19.5	0.433	0.025		0.78	0.306	0.017	1.04 (40)		0.944	0.050		0.07
-17.5	0.770	0.050		0.29	0.116	0.017	1.61 (40)		0.401	0.025		0.84
-15.5	0.551	0.050		0.60	0.496	0.025		0.68	0.286	0.017		1.07
-13.5	0.668	0.050		0.43	0.109	0.017	1.69 (16.5)		0.234	0.025		1.19
-11.5	1.000	0.050		0.00	0.138	0.017		1.48	0.255	0.025		1.14
-9.5	0.851	0.017		0.19	0.957	0.025		0.05	0.958	0.050		0.05
-7.5	0.900	0.050		0.13	0.679	0.025		0.41	0.529	0.017		0.63
-5.5	0.660	0.050		0.44	0.112	0.025		1.59	0.080	0.017		1.75
-3.5	0.892	0.050		0.14	0.566	0.025		0.57	0.517	0.017		0.65
-1.5	0.368	0.017		0.90	0.841	0.050		0.20	0.462	0.025		0.74
0	0.403	0.050		0.84	0.191	0.025		1.31	0.031	0.017		2.15
1.5	0.967	0.050		0.04	0.336	0.017		0.96	0.631	0.025	0.49 (32)	
3.5	0.558	0.017		0.59	0.669	0.025		0.43	0.958	0.050		0.05
5.5	0.826	0.017		0.22	0.862	0.025		0.17	1.000	0.050		0.00
7.5	0.834	0.025		0.21	0.831	0.017		0.21	0.986	0.050		0.02
9.5	0.544	0.017		0.61	0.759	0.050		0.31	0.575	0.025		0.56
21.5	0.773	0.050	0.29 (46)		0.515	0.017	0.66 (40)		0.613	0.025	0.51 (32)	
23.5	0.368	0.025		0.90	0.132	0.017		1.51	0.465	0.050	0.74 (32)	
25.5	0.807	0.050	0.25 (46)		0.439	0.017	0.78 (40)		0.570	0.025	0.57 (32)	
27.5	0.484	0.025		0.70	0.311	0.017		1.01	0.571	0.050	0.57 (32)	
29.5	0.661	0.050		0.44	0.165	0.017		1.39	0.565	0.025	0.58 (32)	

Table S3. Statistics used to assess the inter-group differences of 3-year changes in depth-of-focus (ΔDOF), at each eccentricity (ε). p, probability value; α, significance threshold adjusted using the Holm-Bonferroni method; t, t-statistic for paired t-tests; df, degrees of freedom associated with each t-test; and z, standardized statistic for Wilcoxon tests. * denotes p<α.

ε [deg]	LR vs. HR				MYO vs. LR				MYO vs. HR			
	p	α	|t| (df)	|z|	p	α	|t| (df)	|z|	p	α	|t| (df)	|z|
-29.5	0.256	0.025	1.15 (46)		0.323	0.050		0.99	0.077	0.017		1.77
-27.5	0.804	0.050	0.25 (46)		0.545	0.025	0.61 (40)		0.348	0.017	0.95 (32)	
-25.5	0.959	0.050	0.05 (46)		0.561	0.025	0.59 (40)		0.476	0.017	0.72 (32)	
-23.5	0.784	0.050	0.28 (46)		0.337	0.025	0.97 (38.3)		0.307	0.017	1.04 (29.4)	
-21.5	0.372	0.025	0.9 (46)		0.378	0.050	0.89 (40)		0.086	0.017	1.78 (29)	
-19.5	0.269	0.025	1.12 (46)		0.742	0.050	0.33 (40)		0.183	0.017	1.36 (32)	
-17.5	0.363	0.017	0.92 (46)		0.856	0.050	0.18 (40)		0.546	0.025	0.61 (32)	
-15.5	0.631	0.017		0.48	0.660	0.025		0.44	0.764	0.050	0.3 (32)	
-13.5	0.632	0.025	0.48 (46)		0.308	0.017	1.03 (40)		0.702	0.050	0.39 (30.5)	
-11.5	0.977	0.050	0.03 (29.8)		0.261	0.017	1.14 (40)		0.422	0.025	0.81 (32)	
-9.5	0.827	0.050	0.22 (46)		0.054	0.017	1.98 (40)		0.187	0.025	1.35 (32)	
-7.5	0.992	0.050	0.01 (46)		0.068	0.017	1.88 (40)		0.148	0.025	1.48 (32)	
-5.5	0.702	0.050	0.38 (46)		0.156	0.017	1.45 (40)		0.317	0.025	1.02 (32)	
-3.5	0.584	0.050	0.55 (46)		0.054	0.017	1.98 (40)		0.056	0.025	1.99 (32)	
-1.5	0.699	0.050		0.39	0.280	0.025		1.08	0.247	0.017	1.18 (32)	
0	1.000	0.050		0.00	0.165	0.017		1.39	0.306	0.025	1.04 (32)	
1.5	0.585	0.050	0.55 (46)		0.114	0.025	1.61 (40)		0.077	0.017	1.83 (32)	
3.5	0.818	0.050		0.23	0.153	0.017		1.43	0.161	0.025	1.43 (32)	
5.5	0.714	0.050		0.37	0.235	0.017		1.19	0.307	0.025	1.04 (32)	
7.5	0.645	0.050		0.46	0.364	0.017		0.91	0.424	0.025	0.82 (17.4)	
9.5	0.706	0.025		0.38	0.539	0.017		0.61	0.779	0.050		0.28
21.5	0.092	0.017	1.72 (44.2)		0.768	0.050	0.3 (40)		0.389	0.025	0.88 (17.6)	
23.5	0.061	0.017	1.92 (43.8)		0.225	0.025	1.23 (40)		0.965	0.050	0.04 (17.5)	
25.5	0.593	0.017	0.54 (46)		0.963	0.050	0.05 (40)		0.633	0.025	0.48 (32)	
27.5	0.653	0.025	0.45 (46)		0.632	0.017	0.48 (40)		0.949	0.050	0.06 (32)	
29.5	0.930	0.050	0.09 (46)		0.831	0.025		0.21	0.411	0.017		0.82

Table S4. Statistics used to assess the inter-group differences of final depth-of-focus (DOF_{final}), at each eccentricity (ε). p, probability value; α, significance threshold adjusted using the Holm-Bonferroni method; t, t-statistic for paired t-tests; df, degrees of freedom associated with each t-test; and z, standardized statistic for Wilcoxon tests. * denotes $p<\alpha$.

ε [deg]	LR vs. HR				MYO vs. LR				MYO vs. HR			
	p	α	|t| (df)	|z|	p	α	|t| (df)	|z|	p	α	|t| (df)	|z|
-29.5	0.713	0.050	0.37 (46)		0.708	0.025	0.38 (40)		0.542	0.017	0.62 (32)	
-27.5	0.968	0.050	0.04 (46)		0.580	0.017	0.56 (40)		0.610	0.025	0.51 (32)	
-25.5	0.811	0.050	0.24 (46)		0.546	0.017	0.61 (40)		0.746	0.025	0.33 (32)	
-23.5	0.862	0.050	0.18 (46)		0.330	0.017	0.99 (40)		0.566	0.025	0.58 (32)	
-21.5	0.813	0.050	0.24 (46)		0.313	0.017	1.02 (40)		0.354	0.025	0.94 (32)	
-19.5	0.893	0.050	0.14 (46)		0.281	0.017	1.09 (40)		0.367	0.025	0.91 (32)	
-17.5	0.863	0.050	0.17 (46)		0.314	0.017	1.02 (40)		0.368	0.025	0.91 (32)	
-15.5	0.971	0.050	0.04 (46)		0.287	0.017	1.08 (40)		0.363	0.025	0.92 (32)	
-13.5	0.442	0.050	0.78 (46)		0.010*	0.017	2.71 (40)		0.141	0.025	1.51 (32)	
-11.5	0.714	0.050	0.37 (46)		0.004*	0.017	3.02 (40)		0.050	0.025	2.04 (32)	
-9.5	0.513	0.050	0.66 (27.9)		0.006*	0.017	3.12 (17.4)		0.044	0.025	2.1 (32)	
-7.5	0.912	0.050	0.11 (30.4)		0.023	0.017	2.5 (16.5)		0.031	0.025	2.26 (32)	
-5.5	0.813	0.050	0.24 (46)		0.016*	0.025	2.7 (16.4)		0.015*	0.017	2.56 (32)	
-3.5	0.875	0.050		0.16	0.020*	0.025		2.34	0.006*	0.017	2.95 (32)	
-1.5	0.770	0.050		0.29	0.323	0.025		0.99	0.095	0.017	1.76 (18.5)	
0	0.164	0.050		1.39	0.058	0.025		1.90	0.005*	0.017	3 (32)	
1.5	0.464	0.050	0.74 (46)		0.018*	0.025	2.46 (40)		0.010*	0.017	2.72 (32)	
3.5	0.660	0.050		0.44	0.274	0.025		1.09	0.083	0.017	1.83 (18.9)	
5.5	0.320	0.050		0.99	0.150	0.017		1.44	0.201	0.025	1.33 (16.5)	
7.5	0.254	0.025		1.14	0.098	0.017		1.66	0.344	0.050		0.95
9.5	0.975	0.050		0.03	0.146	0.025		1.45	0.115	0.017	1.66 (18.1)	
21.5	0.112	0.017	1.62 (46)		0.936	0.050		0.08	0.431	0.025		0.79
23.5	0.144	0.017	1.49 (46)		0.619	0.050	0.5 (40)		0.544	0.025	0.61 (32)	
25.5	0.702	0.050	0.39 (46)		0.502	0.025	0.68 (40)		0.324	0.017	1 (32)	
27.5	0.944	0.050	0.07 (46)		0.661	0.025	0.44 (40)		0.643	0.017	0.47 (32)	
29.5	0.735	0.050	0.34 (46)		0.357	0.017	0.93 (40)		0.569	0.025	0.58 (32)	

Table S5. Statistics used to assess the inter-group differences of baseline maximum Strehl ratio ($MaxSR_{bl}$), at each eccentricity (ε). p, probability value; α, significance threshold adjusted using the Holm-Bonferroni method; t, t-statistic for paired t-tests; df, degrees of freedom associated with each t-test; and z, standardized statistic for Wilcoxon tests. * denotes p<α.

ε [deg]	LR vs. HR				MYO vs. LR				MYO vs. HR			
	p	α	|t| (df)	|z|	p	α	|t| (df)	|z|	p	α	|t| (df)	|z|
-29.5	0.310	0.017		1.01	0.959	0.050	0.05 (40)		0.411	0.025		0.82
-27.5	0.310	0.017		1.01	0.739	0.050		0.33	0.662	0.025		0.44
-25.5	0.321	0.017		0.99	0.719	0.025		0.36	0.766	0.050		0.30
-23.5	0.385	0.017		0.87	0.584	0.025		0.55	0.588	0.050		0.54
-21.5	0.310	0.017		1.01	0.463	0.025		0.73	0.793	0.050		0.26
-19.5	0.691	0.025	0.4 (46)		0.416	0.017		0.81	0.875	0.050		0.16
-17.5	0.720	0.050	0.36 (46)		0.186	0.017	1.34 (40)		0.489	0.025	0.7 (32)	
-15.5	0.697	0.050	0.39 (27.5)		0.196	0.017	1.31 (40)		0.253	0.025	1.16 (32)	
-13.5	0.416	0.050	0.83 (24.1)		0.291	0.025	1.09 (18.1)		0.186	0.017	1.35 (32)	
-11.5	0.908	0.050		0.12	0.132	0.017		1.51	0.184	0.025	1.36 (32)	
-9.5	0.971	0.050	0.04 (46)		0.338	0.017	0.97 (40)		0.441	0.025	0.78 (32)	
-7.5	0.692	0.050	0.4 (46)		0.201	0.025	1.3 (40)		0.200	0.017	1.31 (32)	
-5.5	0.425	0.050	0.8 (46)		0.178	0.025	1.37 (40)		0.105	0.017	1.67 (32)	
-3.5	0.684	0.050	0.41 (46)		0.374	0.025	0.9 (40)		0.291	0.017	1.07 (32)	
-1.5	0.321	0.025	1 (46)		0.623	0.050	0.5 (40)		0.254	0.017	1.16 (32)	
0	0.424	0.050	0.81 (46)		0.192	0.025	1.33 (40)		0.059	0.017	1.96 (32)	
1.5	0.812	0.050	0.24 (46)		0.352	0.025	0.94 (40)		0.285	0.017	1.09 (32)	
3.5	0.584	0.017	0.55 (46)		0.964	0.050	0.04 (40)		0.594	0.025	0.54 (32)	
5.5	0.859	0.017	0.18 (46)		0.947	0.050		0.07	0.930	0.025		0.09
7.5	0.962	0.050	0.05 (46)		0.647	0.017	0.46 (40)		0.740	0.025	0.33 (32)	
9.5	0.689	0.025	0.4 (46)		0.789	0.050	0.27 (40)		0.630	0.017	0.49 (32)	
21.5	0.471	0.025		0.72	0.344	0.017		0.95	0.986	0.050		0.02
23.5	0.310	0.025		1.01	0.063	0.017	1.92 (36.3)		0.847	0.050		0.19
25.5	0.471	0.025		0.72	0.257	0.017		1.13	0.495	0.050		0.68
27.5	0.536	0.025	0.62 (46)		0.139	0.017		1.48	0.540	0.050		0.61
29.5	0.498	0.025	0.68 (46)		0.153	0.017		1.43	0.612	0.050		0.51

Table S6. Statistics used to assess the inter-group differences of 3-year changes in maximum Strehl ratio (ΔMaxSR), at each eccentricity (ε). p, probability value; α, significance threshold adjusted using the Holm-Bonferroni method; t, t-statistic for paired t-tests; df, degrees of freedom associated with each t-test; and z, standardized statistic for Wilcoxon tests. * denotes p<α.

ε [deg]	LR vs. HR				MYO vs. LR				MYO vs. HR			
	p	α	|t| (df)	|z|	p	α	|t| (df)	|z|	p	α	|t| (df)	|z|
-29.5	0.119	0.025		1.56	0.321	0.050	1 (40)		0.061	0.017		1.87
-27.5	0.433	0.050		0.78	0.175	0.025	1.38 (40)		0.057	0.017		1.91
-25.5	0.714	0.050		0.37	0.235	0.025		1.19	0.090	0.017		1.70
-23.5	0.565	0.050		0.58	0.246	0.025		1.16	0.083	0.017		1.73
-21.5	0.321	0.025		0.99	0.330	0.050		0.97	0.072	0.017		1.80
-19.5	0.246	0.025		1.16	0.431	0.050		0.79	0.090	0.017		1.70
-17.5	0.458	0.017		0.74	0.780	0.050	0.28 (40)		0.662	0.025		0.44
-15.5	0.908	0.017		0.12	0.968	0.050		0.04	0.919	0.025	0.1 (27.2)	
-13.5	0.975	0.050		0.03	0.841	0.017		0.20	0.864	0.025	0.17 (26.9)	
-11.5	0.714	0.025		0.37	0.431	0.017		0.79	0.989	0.050	0.01 (31.1)	
-9.5	0.753	0.050	0.32 (30.3)		0.454	0.025	0.76 (40)		0.433	0.017	0.79 (32)	
-7.5	0.967	0.050	0.04 (46)		0.767	0.017	0.3 (40)		0.835	0.025	0.21 (31.9)	
-5.5	0.475	0.017	0.72 (46)		0.715	0.025	0.37 (40)		0.770	0.050	0.29 (32)	
-3.5	0.571	0.025	0.57 (46)		0.264	0.017	1.13 (40)		0.644	0.050	0.47 (32)	
-1.5	0.458	0.017		0.74	0.480	0.025		0.71	0.937	0.050	0.08 (32)	
0	0.957	0.050	0.05 (46)		0.728	0.017	0.35 (40)		0.797	0.025	0.26 (32)	
1.5	0.871	0.050	0.16 (46)		0.339	0.025	0.97 (40)		0.306	0.017	1.04 (32)	
3.5	0.868	0.050	0.17 (46)		0.145	0.017	1.49 (40)		0.181	0.025	1.37 (32)	
5.5	0.206	0.025		1.27	0.125	0.017		1.53	0.534	0.050	0.63 (32)	
7.5	0.605	0.050	0.52 (46)		0.232	0.017	1.21 (40)		0.377	0.025	0.9 (32)	
9.5	0.594	0.050		0.53	0.270	0.017	1.12 (40)		0.452	0.025		0.75
21.5	0.041	0.017	2.11 (44.4)		0.431	0.050	0.79 (40)		0.342	0.025	0.97 (22.6)	
23.5	0.037	0.017	2.16 (43.5)		0.140	0.025	1.5 (40)		0.925	0.050	0.09 (32)	
25.5	0.681	0.025	0.41 (46)		0.583	0.017	0.55 (40)		0.829	0.050	0.22 (32)	
27.5	0.572	0.025	0.57 (46)		0.403	0.017	0.85 (40)		0.739	0.050	0.34 (32)	
29.5	0.908	0.050		0.12	0.416	0.025		0.81	0.241	0.017		1.17

Table S7. Statistics used to assess the inter-group differences of final maximum Strehl ratio ($MaxSR_{final}$), at each eccentricity (ε). p, probability value; α, significance threshold adjusted using the Holm-Bonferroni method; t, t-statistic for paired t-tests; df, degrees of freedom associated with each t-test; and z, standardized statistic for Wilcoxon tests. * denotes p<α.

ε [deg]	LR vs. HR				MYO vs. LR				MYO vs. HR			
	p	α	|t| (df)	|z|	p	α	|t| (df)	|z|	p	α	|t| (df)	|z|
-29.5	0.843	0.050		0.20	0.270	0.017	1.12 (40)		0.431	0.025		0.79
-27.5	0.925	0.050		0.09	0.199	0.017	1.31 (40)		0.354	0.025		0.93
-25.5	0.859	0.050		0.18	0.111	0.017	1.63 (37.7)		0.564	0.025		0.58
-23.5	0.699	0.050		0.39	0.137	0.017	1.52 (40)		0.564	0.025		0.58
-21.5	0.958	0.050		0.05	0.225	0.017		1.21	0.391	0.025		0.86
-19.5	1.000	0.050		0.00	0.187	0.017		1.32	0.286	0.025		1.07
-17.5	0.875	0.050		0.16	0.344	0.025		0.95	0.302	0.017		1.03
-15.5	0.810	0.050		0.24	0.161	0.017		1.40	0.319	0.025		1.00
-13.5	0.826	0.050		0.22	0.062	0.017	1.92 (40)		0.146	0.025		1.45
-11.5	0.756	0.050	0.31 (46)		0.039	0.017	2.14 (40)		0.063	0.025	1.93 (32)	
-9.5	0.875	0.050		0.16	0.006*	0.017	2.91 (40)		0.066	0.025		1.84
-7.5	0.958	0.050		0.05	0.016*	0.017	2.53 (40)		0.096	0.025		1.66
-5.5	0.551	0.050		0.60	0.020	0.017		2.33	0.104	0.025		1.63
-3.5	0.551	0.050		0.60	0.008*	0.017		2.65	0.025	0.025	2.35 (32)	
-1.5	0.497	0.050		0.68	0.095	0.017		1.67	0.128	0.025		1.52
0	0.638	0.050		0.47	0.040	0.025	2.13 (40)		0.015*	0.017		2.43
1.5	0.942	0.050		0.07	0.010*	0.017	2.69 (40)		0.031	0.025		2.15
3.5	0.925	0.050		0.09	0.049	0.017	2.03 (40)		0.072	0.025		1.80
5.5	0.213	0.050		1.24	0.156	0.025	1.45 (40)		0.072	0.017		1.80
7.5	0.213	0.050		1.24	0.043	0.017	2.09 (40)		0.111	0.025		1.59
9.5	0.810	0.050	0.24 (46)		0.058	0.017	1.96 (40)		0.118	0.025	1.61 (32)	
21.5	0.205	0.025	1.29 (46)		0.689	0.050	0.4 (40)		0.176	0.017	1.38 (32)	
23.5	0.206	0.017		1.27	0.904	0.050		0.12	0.517	0.025		0.65
25.5	0.875	0.050		0.16	0.603	0.017		0.52	0.662	0.025		0.44
27.5	0.975	0.050		0.03	0.386	0.017		0.87	0.612	0.025		0.51
29.5	0.363	0.017		0.91	0.513	0.025		0.65	0.793	0.050		0.26

Table S8. Statistics used to assess the inter-group differences of baseline center of mass (COM_{bl}), at each eccentricity (ε). p, probability value; α, significance threshold adjusted using the Holm-Bonferroni method; t, t-statistic for paired t-tests; df, degrees of freedom associated with each t-test; and z, standardized statistic for Wilcoxon tests. * denotes p<α.

ε [deg]	LR vs. HR				MYO vs. LR				MYO vs. HR			
	p	α	|t| (df)	|z|	p	α	|t| (df)	|z|	p	α	|t| (df)	|z|
-29.5	0.041	0.017	2.1 (46)		0.363	0.025	0.92 (40)		0.409	0.050	0.84 (32)	
-27.5	0.056	0.017	1.96 (46)		0.479	0.050	0.71 (40)		0.362	0.025	0.92 (32)	
-25.5	0.067	0.017	1.88 (46)		0.492	0.050	0.69 (40)		0.376	0.025	0.9 (32)	
-23.5	0.064	0.017	1.9 (46)		0.388	0.025	0.87 (40)		0.433	0.050	0.79 (32)	
-21.5	0.128	0.017	1.55 (46)		0.409	0.025	0.83 (40)		0.560	0.050	0.59 (32)	
-19.5	0.141	0.017	1.5 (46)		0.460	0.025	0.75 (40)		0.539	0.050	0.62 (32)	
-17.5	0.290	0.017	1.07 (46)		0.306	0.025	1.04 (40)		0.923	0.050	0.1 (32)	
-15.5	0.254	0.025	1.15 (46)		0.240	0.017	1.19 (40)		0.948	0.050	0.07 (32)	
-13.5	0.463	0.025	0.74 (46)		0.159	0.017	1.43 (40)		0.626	0.050	0.49 (32)	
-11.5	0.331	0.025	0.98 (46)		0.040	0.017	2.12 (40)		0.450	0.050	0.77 (32)	
-9.5	0.599	0.050	0.53 (46)		0.057	0.017	1.96 (40)		0.361	0.025	0.93 (32)	
-7.5	0.696	0.050	0.39 (28.6)		0.022	0.017	2.39 (40)		0.205	0.025	1.29 (32)	
-5.5	0.489	0.050	0.7 (46)		0.233	0.025	1.23 (18.6)		0.139	0.017	1.52 (32)	
-3.5	0.681	0.050	0.41 (46)		0.096	0.017	1.75 (19.6)		0.226	0.025	1.23 (32)	
-1.5	0.831	0.050	0.21 (46)		0.082	0.017	1.78 (40)		0.126	0.025	1.57 (32)	
0	0.730	0.017		0.35	0.758	0.025	0.31 (40)		0.958	0.050		0.05
1.5	0.787	0.050	0.27 (46)		0.208	0.017	1.28 (40)		0.245	0.025	1.18 (32)	
3.5	0.158	0.017		1.41	0.904	0.050		0.12	0.193	0.025	1.33 (32)	
5.5	0.105	0.017		1.62	0.401	0.025		0.84	0.431	0.050		0.79
7.5	0.110	0.017		1.60	0.268	0.025		1.11	0.960	0.050	0.05 (32)	
9.5	0.385	0.025		0.87	0.161	0.017		1.40	0.454	0.050	0.76 (32)	
21.5	0.165	0.017	1.41 (46)		0.217	0.025	1.25 (40)		0.922	0.050	0.1 (32)	
23.5	0.933	0.050	0.08 (46)		0.197	0.017	1.31 (40)		0.340	0.025	0.97 (32)	
25.5	0.883	0.050	0.15 (46)		0.236	0.017	1.2 (40)		0.390	0.025	0.87 (32)	
27.5	0.849	0.050	0.19 (46)		0.346	0.017	0.95 (40)		0.535	0.025	0.63 (32)	
29.5	0.672	0.025	0.43 (46)		0.406	0.017	0.84 (40)		0.735	0.050	0.34 (32)	

Table S9. Statistics used to assess the inter-group differences of 3-year changes in center of mass (ΔCOM), at each eccentricity (ε). p, probability value; α, significance threshold adjusted using the Holm-Bonferroni method; t, t-statistic for paired t-tests; df, degrees of freedom associated with each t-test; and z, standardized statistic for Wilcoxon tests. * denotes p<α.

ε [deg]	LR vs. HR				MYO vs. LR				MYO vs. HR			
	p	α	$\lvert t \rvert$ (df)	$\lvert z \rvert$	p	α	$\lvert t \rvert$ (df)	$\lvert z \rvert$	p	α	$\lvert t \rvert$ (df)	$\lvert z \rvert$
-29.5	0.843	0.050		0.20	0.004*	0.017	3.1 (40)		0.052	0.025		1.94
-27.5	0.875	0.050		0.16	0.009*	0.017	2.92 (18.4)		0.034	0.025		2.12
-25.5	0.810	0.050		0.24	0.007*	0.025		2.71	0.007*	0.017		2.71
-23.5	0.975	0.050		0.03	0.013*	0.017		2.49	0.034	0.025		2.12
-21.5	0.653	0.050		0.45	3.9×10^{-4}*	0.017	3.87 (40)		0.029	0.025		2.19
-19.5	0.925	0.050		0.09	0.001	0.017	3.53 (40)		0.018*	0.025		2.36
-17.5	0.925	0.050		0.09	4.0×10^{-4}*	0.017	3.86 (40)		0.029	0.025		2.19
-15.5	0.908	0.050		0.12	0.002*	0.017	3.28 (40)		0.052	0.025		1.94
-13.5	0.958	0.050		0.05	0.007*	0.017	2.84 (40)		0.044	0.025		2.01
-11.5	0.778	0.050		0.28	0.034	0.017	2.2 (40)		0.048	0.025		1.98
-9.5	0.836	0.050	0.21 (46)		0.021	0.017	2.4 (40)		0.119	0.025	1.6 (32)	
-7.5	0.810	0.050		0.24	0.230	0.017	1.22 (40)		0.336	0.025		0.96
-5.5	0.594	0.050		0.53	0.107	0.017	1.65 (40)		0.588	0.025		0.54
-3.5	0.908	0.050		0.12	0.651	0.025	0.46 (40)		0.564	0.017		0.58
-1.5	0.730	0.017		0.35	0.989	0.050		0.01	0.875	0.025		0.16
0	0.638	0.017		0.47	0.831	0.050	0.22 (40)		0.766	0.025		0.30
1.5	0.458	0.050		0.74	0.026	0.017	2.31 (40)		0.167	0.025		1.38
3.5	0.363	0.025		0.91	0.699	0.050		0.39	0.270	0.017		1.10
5.5	0.101	0.017		1.64	0.584	0.050		0.55	0.351	0.025	0.95 (32)	
7.5	0.191	0.017		1.31	0.883	0.050		0.15	0.286	0.025		1.07
9.5	0.565	0.050		0.58	0.496	0.025		0.68	0.227	0.017		1.21
21.5	0.450	0.025	0.76 (46)		0.488	0.050	0.7 (40)		0.128	0.017	1.56 (32)	
23.5	0.714	0.025		0.37	0.856	0.050	0.18 (40)		0.473	0.017		0.72
25.5	0.433	0.050		0.78	0.342	0.025	0.96 (40)		0.061	0.017		1.87
27.5	0.374	0.050		0.89	0.323	0.025	1 (40)		0.178	0.017		1.35
29.5	0.321	0.050		0.99	0.171	0.017	1.39 (40)		0.178	0.025		1.35

Table S10. Statistics used to assess the inter-group differences of final center of mass (COM_{final}), at each eccentricity (ε). p, probability value; α, significance threshold adjusted using the Holm-Bonferroni method; t, t-statistic for paired t-tests; df, degrees of freedom associated with each t-test; and z, standardized statistic for Wilcoxon tests. * denotes p<α.

ε [deg]	LR vs. HR				MYO vs. LR				MYO vs. HR			
	p	α	|t| (df)	|z|	p	α	|t| (df)	|z|	p	α	|t| (df)	|z|
-29.5	0.148	0.025	1.48 (29)		0.003*	0.017	3.2 (40)		0.277	0.050	1.11 (32)	
-27.5	0.131	0.025	1.54 (46)		0.002*	0.017	3.35 (40)		0.174	0.050	1.39 (32)	
-25.5	0.166	0.050	1.41 (46)		0.001*	0.017	3.42 (40)		0.074	0.025	1.84 (32)	
-23.5	0.112	0.050	1.62 (46)		0.001*	0.017	3.45 (40)		0.098	0.025	1.71 (32)	
-21.5	0.189	0.050	1.33 (46)		5.5 $\times10^{-4}$*	0.017	3.76 (40)		0.050	0.025	2.03 (32)	
-19.5	0.371	0.050	0.9 (46)		0.004*	0.017	3.2 (20.2)		0.046	0.025	2.08 (32)	
-17.5	0.493	0.050	0.69 (46)		1.7 $\times10^{-4}$*	0.017	4.14 (40)		0.015*	0.025	2.56 (32)	
-15.5	0.518	0.050	0.65 (46)		2.4 $\times10^{-4}$*	0.017	4.03 (40)		0.022*	0.025	2.41 (32)	
-13.5	0.352	0.050		0.93	7.3 $\times10^{-4}$*	0.017	3.66 (40)		0.044	0.025		2.01
-11.5	0.873	0.050	0.16 (46)		6.2 $\times10^{-4}$*	0.017	3.72 (40)		0.012*	0.025	2.65 (32)	
-9.5	0.789	0.050	0.27 (46)		9.5 $\times10^{-4}$*	0.017	3.57 (40)		0.012*	0.025	2.67 (32)	
-7.5	0.471	0.050		0.72	0.013*	0.017		2.49	0.048	0.025		1.98
-5.5	0.301	0.050		1.04	0.009*	0.017	2.75 (40)		0.037	0.025		2.08
-3.5	0.510	0.050		0.66	0.034	0.017		2.12	0.083	0.025		1.73
-1.5	0.565	0.050		0.58	0.146	0.017		1.45	0.227	0.025		1.21
0	0.536	0.017	0.62 (46)		0.968	0.050	0.04 (40)		0.692	0.025	0.4 (32)	
1.5	0.794	0.050		0.26	0.205	0.017		1.27	0.473	0.025		0.72
3.5	0.683	0.050		0.41	0.285	0.017	1.1 (16.9)		0.637	0.025		0.47
5.5	0.826	0.050		0.22	0.419	0.017	0.83 (17.1)		0.612	0.025		0.51
7.5	0.638	0.050		0.47	0.124	0.017	1.62 (16.5)		0.336	0.025		0.96
9.5	0.221	0.050		1.22	0.030	0.017		2.17	0.202	0.025		1.28
21.5	0.942	0.050		0.07	0.139	0.017		1.48	0.227	0.025		1.21
23.5	0.229	0.017		1.20	0.298	0.025	1.05 (40)		1.000	0.050		0.00
25.5	0.051	0.017		1.96	0.344	0.025		0.95	0.637	0.050		0.47
27.5	0.061	0.017		1.87	0.304	0.025		1.03	0.309	0.050	1.03 (32)	
29.5	0.053	0.017		1.93	0.371	0.025		0.89	0.431	0.050		0.79

Table S11. Statistics used to assess the inter-group differences of baseline logarithmic optical anisotropy ($Log_{10}(OAn_{bl})$), at each eccentricity (ε). p, probability value; α, significance threshold adjusted using the Holm-Bonferroni method; t, t-statistic for paired t-tests; df, degrees of freedom associated with each t-test; and z, standardized statistic for Wilcoxon tests. * denotes p<α.

ε [deg]	LR vs. HR				MYO vs. LR				MYO vs. HR			
	p	α	|t| (df)	|z|	p	α	|t| (df)	|z|	p	α	|t| (df)	|z|
-29.5	0.242	0.017	1.19 (46)		0.425	0.025	0.81 (40)		0.813	0.050	0.24 (32)	
-27.5	0.244	0.017	1.18 (46)		0.581	0.025	0.56 (40)		0.688	0.050	0.4 (32)	
-25.5	0.147	0.017	1.47 (46)		0.664	0.050	0.44 (40)		0.507	0.025	0.67 (32)	
-23.5	0.171	0.017		1.37	0.989	0.050		0.01	0.494	0.025	0.69 (32)	
-21.5	0.291	0.017		1.06	0.647	0.050	0.46 (40)		0.495	0.025		0.68
-19.5	0.244	0.017	1.18 (46)		0.862	0.050	0.17 (40)		0.477	0.025	0.72 (32)	
-17.5	0.380	0.017	0.89 (46)		0.501	0.025	0.68 (40)		0.928	0.050	0.09 (32)	
-15.5	0.421	0.025		0.81	0.401	0.017		0.84	0.741	0.050	0.33 (32)	
-13.5	0.609	0.050	0.51 (46)		0.242	0.017	1.19 (40)		0.547	0.025	0.61 (32)	
-11.5	0.807	0.050	0.25 (46)		0.111	0.017	1.63 (40)		0.243	0.025	1.19 (32)	
-9.5	0.657	0.050	0.45 (46)		0.063	0.017	1.91 (40)		0.070	0.025	1.88 (32)	
-7.5	0.222	0.050	1.24 (46)		0.119	0.025		1.56	0.077	0.017		1.77
-5.5	0.471	0.050		0.72	0.224	0.017	1.23 (40)		0.227	0.025		1.21
-3.5	0.458	0.025		0.74	0.566	0.050		0.57	0.087	0.017	1.77 (32)	
-1.5	0.794	0.050		0.26	0.494	0.025	0.69 (40)		0.431	0.017		0.79
0	0.859	0.050		0.18	0.139	0.025		1.48	0.044	0.017		2.01
1.5	0.626	0.050	0.49 (46)		0.346	0.025	0.95 (40)		0.191	0.017	1.34 (32)	
3.5	0.842	0.050	0.2 (46)		0.573	0.025	0.57 (40)		0.446	0.017	0.77 (32)	
5.5	0.889	0.050	0.14 (46)		0.069	0.017	1.87 (40)		0.084	0.025	1.78 (32)	
7.5	0.947	0.050	0.07 (46)		0.232	0.017	1.21 (40)		0.286	0.025	1.09 (32)	
9.5	0.463	0.025	0.74 (46)		0.634	0.050	0.48 (40)		0.322	0.017	1.01 (32)	
21.5	0.718	0.050	0.36 (46)		0.276	0.017	1.1 (40)		0.549	0.025	0.61 (32)	
23.5	0.567	0.017	0.58 (46)		0.633	0.025	0.48 (40)		0.918	0.050	0.1 (32)	
25.5	0.835	0.050	0.21 (46)		0.828	0.025	0.22 (40)		0.742	0.017	0.33 (32)	
27.5	0.948	0.017	0.07 (46)		0.989	0.050	0.01 (40)		0.972	0.025	0.04 (32)	
29.5	0.508	0.017	0.67 (46)		0.787	0.025	0.27 (40)		0.819	0.050	0.23 (32)	

Table S12. Statistics used to assess the inter-group differences of 3-year changes in logarithmic optical anisotropy ($\Delta Log_{10}(OAn)$), at each eccentricity (ε). p, probability value; α, significance threshold adjusted using the Holm-Bonferroni method; t, t-statistic for paired t-tests; df, degrees of freedom associated with each t-test; and z, standardized statistic for Wilcoxon tests. * denotes p<α.

ε [deg]	LR vs. HR				MYO vs. LR				MYO vs. HR			
	p	α	$\lvert t \rvert$ (df)	$\lvert z \rvert$	p	α	$\lvert t \rvert$ (df)	$\lvert z \rvert$	p	α	$\lvert t \rvert$ (df)	$\lvert z \rvert$
-29.5	0.302	0.050	1.04 (46)		0.100	0.025	1.69 (40)		0.025	0.017	2.35 (32)	
-27.5	0.778	0.050		0.28	0.054	0.017	1.98 (40)		0.066	0.025		1.84
-25.5	0.579	0.050		0.55	0.038	0.017	2.14 (40)		0.044	0.025		2.01
-23.5	0.584	0.050	0.55 (46)		0.101	0.025	1.68 (40)		0.064	0.017	1.92 (32)	
-21.5	0.787	0.050	0.27 (46)		0.053	0.025	1.99 (40)		0.053	0.017	2.01 (32)	
-19.5	0.746	0.050		0.32	0.041	0.017	2.11 (40)		0.083	0.025		1.73
-17.5	0.955	0.050	0.06 (46)		0.102	0.017	1.67 (40)		0.125	0.025	1.58 (32)	
-15.5	0.730	0.050		0.35	0.196	0.017		1.29	0.338	0.025	0.97 (32)	
-13.5	0.817	0.050	0.23 (46)		0.068	0.017		1.83	0.302	0.025		1.03
-11.5	0.817	0.050	0.23 (46)		0.651	0.017	0.46 (40)		0.811	0.025	0.24 (32)	
-9.5	0.566	0.025	0.58 (46)		0.416	0.017		0.81	0.766	0.050		0.30
-7.5	0.409	0.025	0.83 (46)		0.196	0.017		1.29	0.564	0.050		0.58
-5.5	0.670	0.025	0.43 (46)		0.584	0.017		0.55	0.847	0.050		0.19
-3.5	0.581	0.017	0.56 (46)		0.739	0.050		0.33	0.612	0.025		0.51
-1.5	0.356	0.017	0.93 (46)		0.614	0.025	0.51 (15.3)		0.923	0.050	0.1 (16.5)	
0	0.551	0.025		0.60	0.319	0.017	1.03 (14.9)		0.662	0.050		0.44
1.5	0.810	0.050		0.24	0.472	0.017	0.74 (15.8)		0.588	0.025		0.54
3.5	0.668	0.017		0.43	0.763	0.025	0.31 (16.2)		0.766	0.050		0.30
5.5	0.445	0.017		0.76	0.759	0.050		0.31	0.668	0.025	0.43 (32)	
7.5	0.699	0.017		0.39	0.739	0.025		0.33	0.916	0.050	0.11 (32)	
9.5	0.908	0.050		0.12	0.803	0.025	0.25 (40)		0.766	0.017		0.30
21.5	0.301	0.017		1.04	0.373	0.025	0.91 (18.1)		0.540	0.050		0.61
23.5	0.730	0.017		0.35	0.913	0.025	0.11 (18.4)		0.986	0.050		0.02
25.5	0.128	0.017	1.55 (46)		0.574	0.025	0.57 (16.2)		0.791	0.050	0.27 (32)	
27.5	0.099	0.017	1.68 (46)		0.567	0.025	0.59 (15.6)		0.847	0.050	0.2 (16.6)	
29.5	0.409	0.017	0.83 (46)		0.821	0.050		0.23	0.713	0.025		0.37

Table S13. Statistics used to assess the inter-group differences of final logarithmic optical anisotropy ($\text{Log}_{10}(\text{OAn}_{\text{final}})$), at each eccentricity (ε). p, probability value; α, significance threshold adjusted using the Holm-Bonferroni method; t, t-statistic for paired t-tests; df, degrees of freedom associated with each t-test; and z, standardized statistic for Wilcoxon tests. * denotes $p<\alpha$.

ε [deg]	LR vs. HR				MYO vs. LR				MYO vs. HR			
	p	α	|t| (df)	|z|	p	α	|t| (df)	|z|	p	α	|t| (df)	|z|
-29.5	0.640	0.050	0.47 (46)		0.033	0.017	2.21 (40)		0.130	0.025	1.56 (32)	
-27.5	0.508	0.050	0.67 (46)		0.026	0.017	2.32 (40)		0.141	0.025	1.51 (32)	
-25.5	0.504	0.050	0.67 (46)		0.034	0.017		2.12	0.214	0.025		1.24
-23.5	0.247	0.025	1.17 (46)		0.026	0.017	2.31 (40)		0.251	0.050	1.17 (32)	
-21.5	0.190	0.025	1.33 (46)		0.027	0.017	2.41 (17.9)		0.191	0.050	1.34 (32)	
-19.5	0.217	0.050	1.25 (46)		0.032	0.017	2.32 (18.3)		0.216	0.025	1.26 (32)	
-17.5	0.347	0.050	0.95 (46)		0.031	0.017	2.34 (17.3)		0.132	0.025	1.55 (32)	
-15.5	0.460	0.050	0.75 (46)		0.047	0.017	2.13 (18.4)		0.139	0.025	1.52 (32)	
-13.5	0.388	0.050	0.87 (46)		0.018	0.017	2.46 (40)		0.138	0.025	1.52 (32)	
-11.5	0.588	0.050	0.55 (46)		0.043	0.017	2.09 (40)		0.148	0.025	1.48 (32)	
-9.5	0.847	0.050	0.19 (46)		0.143	0.017	1.5 (40)		0.262	0.025	1.14 (32)	
-7.5	0.826	0.050	0.22 (46)		0.218	0.017	1.25 (40)		0.244	0.025	1.19 (32)	
-5.5	0.360	0.050	0.93 (46)		0.225	0.025		1.21	0.146	0.017		1.45
-3.5	0.045	0.017	2.06 (46)		0.317	0.050		1.00	0.104	0.025		1.63
-1.5	0.045	0.017	2.06 (46)		0.814	0.050	0.24 (15.8)		0.475	0.025	0.73 (16.8)	
0	0.296	0.017	1.06 (46)		0.559	0.025	0.6 (14.7)		0.895	0.050	0.13 (17)	
1.5	0.374	0.017		0.89	0.737	0.025	0.34 (15.5)		0.847	0.050		0.19
3.5	0.992	0.050		0.01	0.883	0.017		0.15	0.903	0.025		0.12
5.5	0.443	0.017	0.77 (46)		0.818	0.050	0.23 (15.4)		0.518	0.025	0.65 (32)	
7.5	0.753	0.050	0.32 (46)		0.569	0.025	0.58 (15.5)		0.488	0.017	0.71 (17.2)	
9.5	0.594	0.017		0.53	0.598	0.025	0.54 (16.2)		0.637	0.050		0.47
21.5	0.484	0.025		0.70	0.225	0.017		1.21	0.982	0.050	0.02 (32)	
23.5	0.291	0.017	1.07 (46)		0.790	0.050	0.27 (40)		0.672	0.025	0.43 (32)	
25.5	0.198	0.017		1.29	0.246	0.025		1.16	0.989	0.050	0.01 (32)	
27.5	0.164	0.017		1.39	0.169	0.025		1.37	0.903	0.050		0.12
29.5	0.117	0.017	1.6 (46)		0.455	0.025	0.76 (16.9)		0.782	0.050	0.28 (32)	

2.2 Intra-group baseline-final differences in the through-focus image quality parameters

Table S14. Statistics used to assess the intra-group baseline-final differences in depth-of-focus (DOF) at each eccentricity ε. p, probability value; t, t-statistic for paired t-tests; df, degrees of freedom associated with each t-test; and z, standardized statistic for Wilcoxon tests. * denotes p<0.05.

ε [deg]	LR			HR			MYO		
	p	|t| (df)	|z|	p	|t| (df)	|z|	p	|t| (df)	|z|
-29.5	0.009*	2.72 (54)		0.329	0.99 (38)		0.011*		2.54
-27.5	0.042*	2.08 (54)		0.171	1.4 (38)		0.015*	2.62 (26)	
-25.5	0.039*	2.12 (54)		0.151	1.47 (38)		0.018*	2.52 (26)	
-23.5	0.005*	2.95 (54)		0.132	1.54 (38)		0.003*	3.31 (26)	
-21.5	0.002*	3.33 (54)		0.210	1.28 (38)		0.001*	3.57 (26)	
-19.5	2.1×10^{-5}*	4.66 (54)		0.128	1.56 (38)		0.001*	3.56 (26)	
-17.5	8.9×10^{-6}*	4.91 (54)		0.065	1.9 (38)		0.009*	2.83 (26)	
-15.5	2.6×10^{-4}*		3.65	0.024*	2.35 (38)		0.019*	2.51 (26)	
-13.5	7.2×10^{-4}*	3.59 (54)		0.023*	2.36 (38)		0.017*	2.54 (26)	
-11.5	6.7×10^{-4}*	3.61 (54)		0.038*	2.15 (38)		0.010*	2.77 (26)	
-9.5	5.6×10^{-4}*	3.67 (54)		0.026*	2.32 (38)		0.003*	3.22 (26)	
-7.5	3.3×10^{-4}*	3.84 (54)		0.018*	2.46 (38)		0.003*	3.3 (26)	
-5.5	0.040*	2.11 (54)		0.033*	2.22 (38)		0.021*	2.45 (26)	
-3.5	0.032*	2.2 (54)		0.236	1.21 (38)		0.003*	3.25 (26)	
-1.5	0.155		1.42	0.458	0.75 (38)		0.074	1.86 (26)	
0	0.260		1.13	0.568	0.58 (38)		0.091	1.76 (26)	
1.5	0.287	1.08 (54)		0.810	0.24 (38)		0.036*	2.21 (26)	
3.5	0.900		0.13	0.913	0.11 (38)		0.134	1.55 (26)	
5.5	0.701		0.38	0.665	0.44 (38)		0.203	1.31 (26)	
7.5	0.973		0.03	0.852	0.19 (38)		0.299	1.06 (26)	
9.5	0.640		0.47	0.198		1.29	0.159	1.45 (26)	
21.5	0.031*	2.21 (54)		0.747	0.32 (38)		0.241	1.2 (26)	
23.5	0.057	1.95 (54)		0.820	0.23 (38)		0.927	0.09 (26)	
25.5	0.955	0.06 (54)		0.486	0.7 (38)		0.934	0.08 (26)	
27.5	0.981	0.02 (54)		0.612	0.51 (38)		0.657	0.45 (26)	
29.5	0.666	0.43 (54)		0.663	0.44 (38)		0.245		1.16

Table S15. Statistics used to assess the intra-group baseline-final differences in maximum Strehl ratio (MaxSR) at each eccentricity ε. p, probability value; t, t-statistic for paired t-tests; df, degrees of freedom associated with each t-test; and z, standardized statistic for Wilcoxon tests. * denotes p<0.05.

ε [deg]	LR			HR			MYO		
	p	|t| (df)	|z|	p	|t| (df)	|z|	p	|t| (df)	|z|
-29.5	0.019*	2.42 (54)		0.526		0.63	0.011*	2.73 (26)	
-27.5	0.049*	2.01 (54)		0.145		1.46	0.012*	2.7 (26)	
-25.5	0.048*	2.02 (54)		0.067		1.83	9.8×10^{-4}*		3.30
-23.5	0.012*	2.62 (54)		0.108		1.61	9.8×10^{-4}*		3.30
-21.5	0.007*	2.79 (54)		0.093		1.68	9.8×10^{-4}*		3.30
-19.5	0.004*	3.03 (54)		0.108		1.61	0.001*		3.23
-17.5	4.4×10^{-4}*	3.75 (54)		0.057		1.90	0.017*	2.55 (26)	
-15.5	8.2×10^{-5}*		3.94	0.035*	2.18 (38)		0.008*	2.87 (26)	
-13.5	9.0×10^{-5}*		3.92	0.017*	2.51 (38)		0.008*	2.87 (26)	
-11.5	1.3×10^{-5}*		3.83	0.009*	2.75 (38)		0.008*	2.88 (26)	
-9.5	6.8×10^{-6}*	4.99 (54)		0.022*	2.4 (38)		0.002*	3.52 (26)	
-7.5	1.1×10^{-4}*	4.17 (54)		0.015*	2.54 (38)		0.005*	3.1 (26)	
-5.5	0.015*	2.52 (54)		0.026*	2.32 (38)		0.052	2.04 (26)	
-3.5	0.029*	2.25 (54)		0.027*	2.31 (38)		0.007*	2.9 (26)	
-1.5	0.274		1.09	0.107	1.65 (38)		0.161	1.44 (26)	
0	0.154	1.45 (54)		0.268	1.12 (38)		0.135	1.54 (26)	
1.5	0.169	1.39 (54)		0.414	0.83 (38)		0.031*	2.28 (26)	
3.5	0.735	0.34 (54)		0.663	0.44 (38)		0.081	1.82 (26)	
5.5	0.964		0.05	0.364	0.92 (38)		0.196	1.33 (26)	
7.5	0.735	0.34 (54)		0.393	0.86 (38)		0.097	1.72 (26)	
9.5	0.677	0.42 (54)		0.370		0.90	0.147	1.49 (26)	
21.5	0.017*	2.45 (54)		0.981	0.02 (38)		0.279	1.11 (26)	
23.5	0.098	1.68 (54)		0.526	0.64 (38)		0.605	0.52 (26)	
25.5	0.954	0.06 (54)		0.760	0.31 (38)		0.600	0.53 (26)	
27.5	0.973	0.03 (54)		0.629	0.49 (38)		0.451	0.77 (26)	
29.5	0.925	0.09 (54)		0.654		0.45	0.109		1.60

Table S16. Statistics used to assess the intra-group baseline-final differences in center of mass (COM) at each eccentricity ε. p, probability value; t, t-statistic for paired t-tests; df, degrees of freedom associated with each t-test; and z, standardized statistic for Wilcoxon tests. * denotes p<0.05.

ε [deg]	LR			HR			MYO		
	p	|t| (df)	|z|	p	|t| (df)	|z|	p	|t| (df)	|z|
-29.5	0.066	1.87 (54)		0.048*		1.98	0.011*	2.73 (26)	
-27.5	0.186	1.34 (54)		0.079		1.75	0.008*	2.86 (26)	
-25.5	0.050		1.96	0.135		1.49	0.006*	2.96 (26)	
-23.5	0.255		1.14	0.204		1.27	0.012*	2.69 (26)	
-21.5	0.952	0.06 (54)		0.411		0.82	0.020*	2.48 (26)	
-19.5	0.965	0.04 (54)		1.000		0.00	0.034*	2.24 (26)	
-17.5	0.408	0.83 (54)		0.433		0.78	0.056	2 (26)	
-15.5	0.189	1.33 (54)		0.247		1.16	0.152	1.48 (26)	
-13.5	0.038*	2.12 (54)		0.048*		1.98	0.633	0.48 (26)	
-11.5	0.014*	2.54 (54)		0.028*		2.20	0.929	0.09 (26)	
-9.5	5.9×10^{-4}*	3.65 (54)		0.026*	2.32 (38)		0.674	0.42 (26)	
-7.5	3.3×10^{-4}*	3.83 (54)		0.023*		2.28	0.236	1.21 (26)	
-5.5	3.3×10^{-6}*	5.19 (54)		0.021*		2.31	0.175	1.39 (26)	
-3.5	7.0×10^{-4}*	3.6 (54)		0.007*		2.69	0.107	1.67 (26)	
-1.5	0.053		1.94	0.191		1.31	0.243	1.19 (26)	
0	0.251	1.16 (54)		0.351		0.93	0.432	0.8 (26)	
1.5	0.266	1.12 (54)		0.025*		2.24	0.024*	2.4 (26)	
3.5	0.002*		3.10	0.086		1.72	0.014*	2.65 (26)	
5.5	7.5×10^{-4}*		3.37	0.460	0.75 (38)		0.085	1.79 (26)	
7.5	0.305		1.02	0.839	0.2 (38)		0.875		0.16
9.5	0.111		1.59	0.062		1.87	0.875		0.16
21.5	0.572	0.57 (54)		0.179	1.37 (38)		0.760	0.31 (26)	
23.5	0.024*	2.33 (54)		0.156		1.42	0.099	1.71 (26)	
25.5	0.009*	2.7 (54)		0.079		1.75	0.040*	2.16 (26)	
27.5	0.012*	2.58 (54)		0.232		1.19	0.081	1.82 (26)	
29.5	0.013*	2.57 (54)		0.204		1.27	0.068	1.9 (26)	

Table S17. Statistics used to assess the intra-group baseline-final differences in logarithmic optical anisotropy $Log_{10}(OAn)$ at each eccentricity ε. p, probability value; t, t-statistic for paired t-tests; df, degrees of freedom associated with each t-test; and z, standardized statistic for Wilcoxon tests. * denotes p<0.05.

ε [deg]	LR			HR			MYO		
	p	|t| (df)	|z|	p	|t| (df)	|z|	p	|t| (df)	|z|
-29.5	0.117	1.59 (54)		0.639	0.47 (38)		0.054	2.02 (26)	
-27.5	0.268	1.12 (54)		0.232		1.19	0.061	1.96 (26)	
-25.5	0.348	0.95 (54)		0.654		0.45	0.062	1.95 (26)	
-23.5	0.465	0.74 (54)		0.930	0.09 (38)		0.121	1.6 (26)	
-21.5	0.653	0.45 (54)		0.920	0.1 (38)		0.110	1.65 (26)	
-19.5	0.860	0.18 (54)		0.433		0.78	0.098	1.72 (26)	
-17.5	0.939	0.08 (54)		0.922	0.1 (38)		0.183	1.37 (26)	
-15.5	0.327		0.98	0.575	0.57 (38)		0.564	0.58 (26)	
-13.5	0.157	1.43 (54)		0.411	0.83 (38)		0.433		0.78
-11.5	0.110	1.62 (54)		0.318	1.01 (38)		0.638	0.48 (26)	
-9.5	0.072	1.83 (54)		0.462	0.74 (38)		0.826		0.22
-7.5	0.075	1.82 (54)		0.649	0.46 (38)		0.683		0.41
-5.5	0.310	1.02 (54)		0.811	0.24 (38)		0.925		0.09
-3.5	0.923	0.1 (54)		0.572	0.57 (38)		0.975		0.03
-1.5	0.474	0.72 (54)		0.713	0.37 (38)		0.745	0.33 (26)	
0	0.538	0.62 (54)		0.737		0.34	0.323	1.01 (26)	
1.5	0.887	0.14 (54)		0.881		0.15	0.416	0.83 (26)	
3.5	0.690	0.4 (54)		0.940		0.07	0.840	0.2 (26)	
5.5	0.179		1.34	0.756	0.31 (38)		0.763	0.3 (26)	
7.5	0.202		1.28	0.918	0.1 (38)		0.874	0.16 (26)	
9.5	0.730	0.35 (54)		0.881		0.15	0.938	0.08 (26)	
21.5	0.599	0.53 (54)		0.391		0.86	0.411	0.84 (26)	
23.5	0.509	0.66 (54)		0.351		0.93	0.631	0.49 (26)	
25.5	0.032	2.21 (54)		0.790	0.27 (38)		0.646	0.47 (26)	
27.5	0.010	2.65 (54)		0.693	0.4 (38)		0.650	0.46 (26)	
29.5	0.075	1.82 (54)		0.626	0.49 (38)		0.331		0.97

2.3 Inter-group comparison of linear relationships between baseline through-focus image quality parameters and descriptors of 3-year refractive development

Table S18. Statistics used for the inter-group comparison between baseline depth-of-focus (DOF_{bl}) at each ε and 3-year changes of the negative central spherocylindrical error (-ΔcSE). PCC, Pearson correlation coefficient; p, probability value; α, significance threshold adjusted using the Holm-Bonferroni method. * denotes p<α.

ε [deg]	LR			HR			MYO		
	PCC	p	α	PCC	p	α	PCC	p	α
-29.5	0.220	0.260	0.017	0.261	0.266	0.025	0.122	0.679	0.050
-27.5	0.128	0.516	0.025	0.272	0.246	0.017	-0.050	0.866	0.050
-25.5	0.042	0.831	0.025	0.228	0.333	0.017	-0.021	0.942	0.050
-23.5	0.133	0.499	0.050	0.245	0.297	0.025	-0.429	0.126	0.017
-21.5	0.181	0.356	0.050	0.280	0.232	0.025	-0.466	0.093	0.017
-19.5	0.148	0.453	0.050	0.288	0.218	0.025	-0.521	0.056	0.017
-17.5	0.226	0.246	0.050	0.345	0.136	0.025	-0.423	0.132	0.017
-15.5	0.277	0.154	0.017	0.307	0.187	0.025	-0.147	0.617	0.050
-13.5	0.278	0.153	0.017	0.290	0.214	0.025	-0.223	0.443	0.050
-11.5	0.102	0.606	0.025	0.241	0.306	0.017	-0.119	0.685	0.050
-9.5	0.061	0.759	0.025	0.123	0.605	0.017	0.053	0.857	0.050
-7.5	0.216	0.269	0.025	0.085	0.723	0.050	0.392	0.166	0.017
-5.5	0.098	0.619	0.025	0.108	0.652	0.050	0.517	0.058	0.017
-3.5	0.151	0.443	0.050	-0.186	0.434	0.025	0.478	0.084	0.017
-1.5	0.252	0.196	0.025	-0.285	0.222	0.050	0.455	0.102	0.017
0	0.077	0.695	0.050	-0.258	0.273	0.017	0.301	0.296	0.025
1.5	0.277	0.154	0.025	-0.256	0.275	0.050	0.579	0.030	0.017
3.5	0.053	0.789	0.050	-0.128	0.591	0.025	0.636	0.014*	0.017
5.5	0.208	0.288	0.025	-0.158	0.505	0.050	0.481	0.082	0.017
7.5	0.385	0.043	0.017	-0.235	0.319	0.050	0.427	0.127	0.025
9.5	0.165	0.401	0.050	-0.244	0.301	0.025	0.371	0.191	0.017
21.5	-0.121	0.540	0.025	-0.323	0.165	0.017	0.154	0.599	0.050
23.5	-0.087	0.660	0.050	-0.360	0.119	0.017	-0.348	0.223	0.025
25.5	-0.183	0.351	0.025	-0.222	0.346	0.017	-0.181	0.536	0.050
27.5	-0.064	0.747	0.050	-0.258	0.273	0.017	-0.201	0.491	0.025
29.5	-0.203	0.299	0.017	-0.155	0.513	0.025	-0.002	0.996	0.050

Table S19. Statistics used for the inter-group comparison between baseline depth-of-focus (DOF_{bl}) at each ε and 3-year changes of the axial length to corneal radius ratio (ΔAXL/CR). PCC, Pearson correlation coefficient; p, probability value; α, significance threshold adjusted using the Holm-Bonferroni method. * denotes p<α.

ε [deg]	LR			HR			MYO		
	PCC	p	α	PCC	p	α	PCC	p	α
-29.5	0.227	0.244	0.025	-0.275	0.240	0.017	0.157	0.593	0.050
-27.5	0.133	0.498	0.025	-0.224	0.343	0.017	0.068	0.818	0.050
-25.5	0.052	0.793	0.025	-0.171	0.472	0.017	-0.019	0.948	0.050
-23.5	0.128	0.517	0.025	-0.142	0.551	0.050	-0.495	0.072	0.017
-21.5	0.205	0.296	0.025	-0.085	0.723	0.050	-0.532	0.050	0.017
-19.5	0.245	0.209	0.025	-0.084	0.723	0.050	-0.602	0.023	0.017
-17.5	0.259	0.184	0.025	0.019	0.936	0.050	-0.591	0.026	0.017
-15.5	0.270	0.164	0.017	0.103	0.665	0.050	-0.265	0.361	0.025
-13.5	0.474	0.011*	0.017	0.207	0.382	0.050	-0.400	0.157	0.025
-11.5	0.250	0.199	0.025	0.319	0.171	0.017	-0.278	0.337	0.050
-9.5	0.146	0.458	0.025	0.468	0.038	0.017	0.043	0.883	0.050
-7.5	0.125	0.526	0.050	0.483	0.031	0.017	0.291	0.312	0.025
-5.5	0.139	0.480	0.050	0.493	0.027	0.017	0.316	0.271	0.025
-3.5	0.018	0.930	0.050	0.579	0.008*	0.017	0.349	0.222	0.025
-1.5	-0.173	0.379	0.050	0.514	0.021	0.017	0.279	0.333	0.025
0	0.191	0.331	0.025	0.570	0.009*	0.017	0.100	0.734	0.050
1.5	0.059	0.765	0.050	0.460	0.041	0.017	0.384	0.176	0.025
3.5	-0.077	0.699	0.050	0.567	0.009*	0.017	0.411	0.145	0.025
5.5	0.103	0.603	0.050	0.342	0.140	0.017	0.284	0.326	0.025
7.5	0.017	0.932	0.050	0.231	0.327	0.017	0.223	0.443	0.025
9.5	0.059	0.765	0.050	0.101	0.671	0.025	0.136	0.644	0.017
21.5	-0.103	0.602	0.017	-0.098	0.682	0.025	-0.074	0.800	0.050
23.5	-0.118	0.550	0.050	-0.248	0.291	0.025	-0.317	0.270	0.017
25.5	-0.197	0.316	0.017	-0.228	0.334	0.025	-0.036	0.904	0.050
27.5	-0.245	0.210	0.017	-0.210	0.374	0.025	-0.034	0.907	0.050
29.5	-0.279	0.150	0.017	-0.197	0.405	0.025	0.058	0.844	0.050

Table S20. Statistics used for the inter-group comparison between baseline maximum Strehl ratio ($MaxSR_{bl}$) at each ε and 3-year changes of the negative central spherocylindrical error (-ΔcSE). PCC, Pearson correlation coefficient; p, probability value; α, significance threshold adjusted using the Holm-Bonferroni method. * denotes p<α.

ε [deg]	LR			HR			MYO		
	PCC	p	α	PCC	p	α	PCC	p	α
-29.5	-0.148	0.452	0.050	-0.264	0.260	0.017	0.240	0.408	0.025
-27.5	-0.065	0.741	0.050	-0.232	0.325	0.017	0.237	0.414	0.025
-25.5	-0.066	0.738	0.050	-0.160	0.500	0.025	0.291	0.313	0.017
-23.5	-0.117	0.553	0.050	-0.144	0.544	0.025	0.465	0.094	0.017
-21.5	-0.138	0.485	0.050	-0.171	0.471	0.025	0.469	0.091	0.017
-19.5	-0.191	0.330	0.025	-0.161	0.499	0.050	0.513	0.061	0.017
-17.5	-0.185	0.345	0.025	-0.213	0.367	0.050	0.390	0.168	0.017
-15.5	-0.223	0.255	0.017	-0.148	0.534	0.025	0.136	0.643	0.050
-13.5	-0.243	0.213	0.017	-0.081	0.735	0.050	0.180	0.539	0.025
-11.5	-0.113	0.567	0.050	-0.194	0.413	0.017	0.232	0.426	0.025
-9.5	-0.163	0.407	0.017	-0.061	0.798	0.025	0.024	0.936	0.050
-7.5	-0.311	0.107	0.017	-0.013	0.958	0.050	-0.207	0.478	0.025
-5.5	-0.324	0.092	0.017	0.028	0.908	0.050	-0.307	0.285	0.025
-3.5	-0.374	0.050	0.017	0.126	0.597	0.050	-0.300	0.297	0.025
-1.5	-0.390	0.040	0.017	0.246	0.295	0.050	-0.344	0.229	0.025
0	-0.368	0.054	0.017	0.263	0.262	0.025	-0.241	0.406	0.050
1.5	-0.425	0.024	0.017	0.235	0.320	0.050	-0.373	0.189	0.025
3.5	-0.241	0.217	0.025	0.251	0.287	0.050	-0.487	0.077	0.017
5.5	-0.430	0.022	0.017	0.212	0.369	0.050	-0.459	0.099	0.025
7.5	-0.525	0.004*	0.017	0.259	0.270	0.025	-0.307	0.286	0.050
9.5	-0.244	0.211	0.017	0.223	0.345	0.025	-0.188	0.519	0.050
21.5	0.103	0.601	0.025	0.312	0.180	0.017	-0.034	0.908	0.050
23.5	0.136	0.491	0.050	0.325	0.162	0.017	0.364	0.201	0.025
25.5	0.110	0.577	0.025	0.253	0.282	0.017	0.062	0.834	0.050
27.5	0.040	0.840	0.050	0.229	0.331	0.017	0.106	0.719	0.025
29.5	0.056	0.778	0.025	0.108	0.649	0.017	-0.020	0.947	0.050

Table S21. Statistics used for the inter-group comparison between baseline maximum Strehl ratio ($MaxSR_{bl}$) at each ε and 3-year changes of the axial length to corneal radius ratio (ΔAXL/CR). PCC, Pearson correlation coefficient; p, probability value; α, significance threshold adjusted using the Holm-Bonferroni method. * denotes p<α.

ε [deg]	LR			HR			MYO		
	PCC	p	α	PCC	p	α	PCC	p	α
-29.5	0.018	0.927	0.050	0.439	0.053	0.017	0.152	0.605	0.025
-27.5	0.100	0.613	0.025	0.330	0.156	0.017	0.094	0.749	0.050
-25.5	0.030	0.880	0.050	0.199	0.400	0.025	0.253	0.383	0.017
-23.5	-0.002	0.990	0.050	0.166	0.485	0.025	0.450	0.107	0.017
-21.5	-0.076	0.701	0.025	0.047	0.844	0.050	0.459	0.099	0.017
-19.5	-0.082	0.677	0.050	0.125	0.601	0.025	0.561	0.037	0.017
-17.5	-0.155	0.431	0.025	0.083	0.727	0.050	0.582	0.029	0.017
-15.5	-0.257	0.187	0.017	-0.061	0.798	0.050	0.314	0.275	0.025
-13.5	-0.462	0.013*	0.017	-0.127	0.594	0.050	0.408	0.147	0.025
-11.5	-0.327	0.090	0.025	-0.331	0.154	0.050	0.491	0.075	0.017
-9.5	-0.197	0.314	0.025	-0.452	0.045	0.017	0.168	0.565	0.050
-7.5	-0.167	0.396	0.025	-0.447	0.048	0.017	-0.048	0.870	0.050
-5.5	-0.273	0.159	0.025	-0.447	0.048	0.017	-0.145	0.620	0.050
-3.5	-0.093	0.636	0.050	-0.401	0.080	0.017	-0.214	0.463	0.025
-1.5	0.040	0.841	0.050	-0.393	0.087	0.017	-0.185	0.527	0.025
0	-0.187	0.340	0.025	-0.352	0.128	0.017	0.041	0.888	0.050
1.5	-0.182	0.354	0.025	-0.335	0.149	0.017	-0.195	0.505	0.050
3.5	0.064	0.745	0.050	-0.446	0.048	0.017	-0.287	0.320	0.025
5.5	-0.271	0.163	0.017	-0.303	0.194	0.025	-0.286	0.322	0.050
7.5	-0.229	0.241	0.017	-0.180	0.447	0.025	-0.084	0.776	0.050
9.5	-0.126	0.523	0.017	-0.135	0.570	0.025	0.127	0.666	0.050
21.5	0.035	0.860	0.050	-0.059	0.804	0.025	0.232	0.424	0.017
23.5	-0.024	0.904	0.025	0.022	0.928	0.050	0.344	0.229	0.017
25.5	0.093	0.636	0.017	0.050	0.836	0.025	-0.028	0.924	0.050
27.5	0.089	0.652	0.017	0.064	0.789	0.025	-0.036	0.903	0.050
29.5	0.102	0.604	0.025	0.231	0.328	0.017	-0.143	0.625	0.050

Table S22. Statistics used for the inter-group comparison between baseline center of mass (COM_{bl}) at each ε and 3-year changes of the negative central spherocylindrical error (-ΔcSE). PCC, Pearson correlation coefficient; p, probability value; α, significance threshold adjusted using the Holm-Bonferroni method. * denotes p<α.

ε [deg]	LR			HR			MYO		
	PCC	p	α	PCC	p	α	PCC	p	α
-29.5	0.129	0.515	0.017	0.042	0.861	0.050	-0.186	0.524	0.025
-27.5	0.150	0.446	0.017	0.060	0.802	0.050	-0.157	0.593	0.025
-25.5	0.147	0.455	0.017	0.103	0.666	0.050	-0.164	0.576	0.025
-23.5	0.107	0.588	0.017	0.124	0.602	0.025	-0.080	0.785	0.050
-21.5	0.099	0.617	0.025	0.192	0.418	0.017	-0.111	0.706	0.050
-19.5	0.077	0.698	0.025	0.217	0.357	0.017	-0.097	0.743	0.050
-17.5	0.059	0.767	0.025	0.255	0.279	0.017	-0.055	0.852	0.050
-15.5	0.075	0.703	0.025	0.280	0.232	0.017	-0.093	0.752	0.050
-13.5	0.027	0.890	0.050	0.281	0.230	0.017	-0.283	0.327	0.025
-11.5	0.017	0.932	0.050	0.290	0.215	0.017	-0.198	0.498	0.025
-9.5	0.018	0.926	0.050	0.272	0.246	0.017	-0.205	0.483	0.025
-7.5	-0.044	0.825	0.050	0.274	0.242	0.017	-0.286	0.322	0.025
-5.5	-0.082	0.678	0.025	0.354	0.125	0.017	-0.063	0.830	0.050
-3.5	0.066	0.740	0.050	0.386	0.093	0.017	0.131	0.655	0.025
-1.5	0.099	0.616	0.050	0.229	0.331	0.017	0.229	0.431	0.025
0	-0.046	0.818	0.050	0.455	0.044	0.017	0.208	0.476	0.025
1.5	-0.221	0.258	0.050	0.385	0.094	0.017	0.353	0.215	0.025
3.5	-0.098	0.621	0.025	0.058	0.807	0.050	0.296	0.304	0.017
5.5	-0.184	0.350	0.017	-0.066	0.782	0.025	-0.023	0.937	0.050
7.5	-0.252	0.196	0.017	-0.273	0.243	0.025	0.248	0.393	0.050
9.5	-0.154	0.433	0.050	-0.209	0.375	0.025	0.305	0.290	0.017
21.5	-0.196	0.317	0.025	-0.148	0.533	0.050	0.322	0.262	0.017
23.5	-0.153	0.437	0.025	-0.220	0.352	0.017	0.119	0.685	0.050
25.5	-0.132	0.502	0.017	-0.156	0.512	0.025	0.035	0.907	0.050
27.5	-0.125	0.526	0.025	-0.180	0.447	0.017	0.003	0.993	0.050
29.5	-0.133	0.500	0.017	-0.073	0.761	0.025	-0.018	0.952	0.050

Table S23. Statistics used for the inter-group comparison between baseline center of mass (COM_{bl}) at each ε and 3-year changes of the axial length to corneal radius ratio (ΔAXL/CR). PCC, Pearson correlation coefficient; p, probability value; α, significance threshold adjusted using the Holm-Bonferroni method. * denotes p<α.

ε [deg]	LR			HR			MYO		
	PCC	p	α	PCC	p	α	PCC	p	α
-29.5	0.061	0.758	0.017	0.046	0.847	0.025	-0.051	0.864	0.050
-27.5	0.001	0.995	0.050	0.110	0.644	0.017	0.022	0.939	0.025
-25.5	-0.013	0.947	0.050	0.141	0.554	0.017	0.026	0.929	0.025
-23.5	-0.073	0.714	0.050	0.118	0.620	0.017	0.139	0.636	0.025
-21.5	-0.075	0.706	0.050	0.184	0.437	0.017	0.121	0.681	0.025
-19.5	-0.089	0.653	0.025	0.230	0.329	0.017	0.113	0.700	0.050
-17.5	-0.066	0.740	0.050	0.188	0.429	0.017	0.100	0.733	0.025
-15.5	-0.041	0.836	0.025	0.210	0.374	0.017	-0.031	0.916	0.050
-13.5	-0.008	0.968	0.050	0.205	0.385	0.017	-0.206	0.479	0.025
-11.5	0.003	0.988	0.050	0.171	0.470	0.017	-0.083	0.778	0.025
-9.5	0.088	0.655	0.025	0.160	0.500	0.017	-0.076	0.796	0.050
-7.5	0.180	0.358	0.017	0.079	0.740	0.025	-0.015	0.959	0.050
-5.5	0.241	0.216	0.025	0.290	0.215	0.017	0.250	0.389	0.050
-3.5	0.288	0.138	0.017	0.341	0.141	0.025	0.387	0.171	0.050
-1.5	0.245	0.209	0.050	0.413	0.070	0.017	0.447	0.109	0.025
0	-0.050	0.802	0.050	0.310	0.184	0.025	0.400	0.157	0.017
1.5	0.176	0.371	0.025	0.091	0.702	0.050	0.503	0.067	0.017
3.5	0.263	0.177	0.025	0.112	0.638	0.050	0.416	0.139	0.017
5.5	0.185	0.346	0.025	0.249	0.290	0.017	0.203	0.486	0.050
7.5	0.057	0.775	0.050	0.386	0.093	0.017	0.334	0.242	0.025
9.5	0.088	0.656	0.050	0.383	0.096	0.017	0.282	0.329	0.025
21.5	-0.432	0.022	0.017	0.198	0.403	0.025	0.197	0.500	0.050
23.5	-0.250	0.200	0.017	0.259	0.271	0.025	0.093	0.751	0.050
25.5	-0.179	0.363	0.025	0.260	0.268	0.017	0.090	0.759	0.050
27.5	-0.177	0.368	0.017	0.158	0.507	0.025	0.050	0.865	0.050
29.5	-0.116	0.555	0.017	0.127	0.594	0.025	0.057	0.847	0.050

Table S24. Statistics used for the inter-group comparison between baseline logarithmic optical anisotropy ($Log_{10}(OAn_{bl})$) at each ε and 3-year changes of the negative central spherocylindrical error (-ΔcSE). PCC, Pearson correlation coefficient; p, probability value; α, significance threshold adjusted using the Holm-Bonferroni method. * denotes p<α.

ε [deg]	LR			HR			MYO		
	PCC	p	α	PCC	p	α	PCC	p	α
-29.5	-0.138	0.482	0.025	0.153	0.520	0.050	0.229	0.430	0.017
-27.5	-0.120	0.541	0.025	0.113	0.635	0.050	0.184	0.529	0.017
-25.5	-0.095	0.632	0.025	0.014	0.952	0.050	0.184	0.528	0.017
-23.5	-0.059	0.766	0.025	0.019	0.937	0.050	0.155	0.596	0.017
-21.5	-0.014	0.945	0.025	-0.002	0.993	0.050	0.129	0.661	0.017
-19.5	0.046	0.816	0.050	-0.136	0.566	0.017	0.116	0.694	0.025
-17.5	0.152	0.441	0.017	-0.092	0.699	0.050	0.152	0.605	0.025
-15.5	0.170	0.386	0.017	-0.194	0.413	0.025	0.185	0.527	0.050
-13.5	0.204	0.298	0.017	-0.223	0.345	0.025	0.215	0.459	0.050
-11.5	0.264	0.174	0.017	-0.183	0.440	0.050	0.267	0.356	0.025
-9.5	0.005	0.980	0.050	-0.247	0.295	0.025	0.402	0.154	0.017
-7.5	0.028	0.886	0.050	-0.115	0.629	0.025	0.296	0.304	0.017
-5.5	-0.071	0.718	0.025	-0.148	0.534	0.017	0.065	0.827	0.050
-3.5	-0.164	0.405	0.017	-0.008	0.975	0.050	0.070	0.812	0.025
-1.5	0.083	0.675	0.017	-0.026	0.914	0.050	0.076	0.797	0.025
0	-0.030	0.878	0.050	0.129	0.588	0.025	0.319	0.266	0.017
1.5	-0.081	0.681	0.025	0.149	0.532	0.017	0.019	0.950	0.050
3.5	0.048	0.809	0.050	0.093	0.695	0.025	0.210	0.472	0.017
5.5	-0.093	0.636	0.025	0.046	0.848	0.050	0.375	0.186	0.017
7.5	-0.083	0.675	0.050	-0.158	0.505	0.025	0.452	0.105	0.017
9.5	-0.091	0.644	0.025	0.029	0.904	0.050	0.491	0.075	0.017
21.5	0.165	0.401	0.017	-0.095	0.691	0.025	-0.021	0.943	0.050
23.5	0.184	0.349	0.025	-0.032	0.895	0.050	0.324	0.258	0.017
25.5	0.078	0.692	0.025	0.018	0.940	0.050	0.361	0.204	0.017
27.5	-0.003	0.986	0.050	0.036	0.879	0.025	0.428	0.127	0.017
29.5	0.002	0.990	0.050	0.009	0.969	0.025	0.319	0.267	0.017

Table S25. Statistics used for the inter-group comparison between baseline logarithmic optical anisotropy ($Log_{10}(OAn_{bl})$) at each ε and 3-year changes of the axial length to corneal radius ratio (ΔAXL/CR). PCC, Pearson correlation coefficient; p, probability value; α, significance threshold adjusted using the Holm-Bonferroni method. * denotes p<α.

ε [deg]	LR			HR			MYO		
	PCC	p	α	PCC	p	α	PCC	p	α
-29.5	0.060	0.760	0.025	-0.241	0.307	0.017	0.067	0.821	0.050
-27.5	0.074	0.709	0.025	-0.186	0.432	0.017	-0.007	0.982	0.050
-25.5	0.084	0.672	0.025	-0.145	0.542	0.017	-0.026	0.930	0.050
-23.5	0.101	0.608	0.017	-0.078	0.744	0.025	-0.048	0.872	0.050
-21.5	0.165	0.401	0.017	-0.147	0.537	0.025	-0.008	0.979	0.050
-19.5	0.161	0.414	0.017	-0.053	0.823	0.025	-0.065	0.825	0.050
-17.5	0.298	0.123	0.017	-0.149	0.531	0.025	0.024	0.935	0.050
-15.5	0.405	0.033	0.017	-0.244	0.299	0.025	0.075	0.800	0.050
-13.5	0.363	0.057	0.017	-0.222	0.348	0.025	0.111	0.706	0.050
-11.5	0.380	0.046	0.017	-0.317	0.173	0.025	0.278	0.337	0.050
-9.5	0.258	0.185	0.017	-0.301	0.197	0.025	0.304	0.291	0.050
-7.5	0.255	0.190	0.017	-0.230	0.330	0.025	0.081	0.784	0.050
-5.5	0.216	0.269	0.017	-0.211	0.371	0.025	-0.210	0.471	0.050
-3.5	0.107	0.589	0.050	-0.360	0.119	0.017	-0.177	0.546	0.025
-1.5	0.244	0.210	0.025	-0.382	0.096	0.017	-0.140	0.632	0.050
0	0.037	0.853	0.025	-0.346	0.135	0.017	0.020	0.946	0.050
1.5	0.097	0.623	0.050	-0.374	0.104	0.017	-0.159	0.587	0.025
3.5	0.135	0.494	0.025	-0.474	0.035	0.017	0.091	0.758	0.050
5.5	0.119	0.547	0.050	-0.284	0.224	0.017	0.275	0.342	0.025
7.5	0.015	0.939	0.050	-0.129	0.588	0.025	0.517	0.058	0.017
9.5	0.056	0.775	0.050	0.341	0.142	0.025	0.544	0.045	0.017
21.5	0.189	0.335	0.025	0.421	0.064	0.017	0.190	0.515	0.050
23.5	0.302	0.119	0.017	0.287	0.221	0.025	0.299	0.300	0.050
25.5	0.052	0.792	0.050	0.250	0.287	0.017	0.208	0.475	0.025
27.5	0.101	0.610	0.050	0.227	0.336	0.017	0.255	0.378	0.025
29.5	-0.034	0.864	0.050	0.129	0.588	0.025	0.162	0.580	0.017

2.4 Inter-group comparison of linear relationships between 3-year change in through-focus image quality parameters and descriptors of 3-year refractive development

Table S26. Statistics used for the inter-group comparison between 3-year changes of depth-of-focus (ΔDOF) at each ε and 3-year changes of the negative central spherocylindrical error (-ΔcSE). PCC, Pearson correlation coefficient; p, probability value; α, significance threshold adjusted using the Holm-Bonferroni method. * denotes p<α.

ε [deg]	LR			HR			MYO		
	PCC	p	α	PCC	p	α	PCC	p	α
-29.5	-0.280	0.149	0.017	-0.266	0.256	0.025	-0.037	0.901	0.050
-27.5	-0.059	0.764	0.025	-0.164	0.490	0.017	0.077	0.794	0.050
-25.5	0.129	0.513	0.017	0.030	0.901	0.050	-0.134	0.648	0.025
-23.5	0.134	0.498	0.025	0.052	0.827	0.050	0.305	0.290	0.017
-21.5	-0.105	0.595	0.025	0.067	0.780	0.050	0.575	0.031	0.017
-19.5	-0.056	0.776	0.050	-0.105	0.659	0.025	0.658	0.011*	0.017
-17.5	-0.169	0.390	0.025	-0.201	0.396	0.050	0.614	0.019	0.017
-15.5	-0.235	0.228	0.025	-0.206	0.383	0.050	0.472	0.089	0.017
-13.5	-0.244	0.210	0.025	-0.115	0.628	0.050	0.548	0.042	0.017
-11.5	-0.346	0.071	0.017	-0.127	0.594	0.050	0.414	0.141	0.025
-9.5	-0.264	0.174	0.017	-0.172	0.469	0.025	0.125	0.671	0.050
-7.5	-0.331	0.085	0.017	-0.042	0.860	0.025	0.038	0.898	0.050
-5.5	-0.276	0.155	0.017	-0.044	0.852	0.050	-0.086	0.771	0.025
-3.5	-0.361	0.059	0.017	0.157	0.508	0.025	-0.036	0.903	0.050
-1.5	-0.359	0.060	0.017	0.207	0.380	0.025	-0.168	0.567	0.050
0	-0.247	0.206	0.017	0.282	0.229	0.025	-0.007	0.981	0.050
1.5	-0.466	0.012*	0.017	0.156	0.512	0.025	-0.055	0.852	0.050
3.5	-0.207	0.290	0.017	0.100	0.676	0.025	-0.078	0.791	0.050
5.5	-0.194	0.323	0.017	0.196	0.407	0.025	0.122	0.678	0.050
7.5	-0.249	0.201	0.017	0.174	0.463	0.025	0.110	0.708	0.050
9.5	-0.072	0.716	0.050	0.394	0.085	0.017	0.140	0.633	0.025
21.5	-0.029	0.885	0.050	0.194	0.412	0.025	0.481	0.081	0.017
23.5	0.022	0.910	0.050	0.287	0.220	0.025	0.667	0.009*	0.017
25.5	0.190	0.332	0.025	0.121	0.610	0.050	0.600	0.023	0.017
27.5	-0.044	0.824	0.050	0.156	0.510	0.025	0.488	0.076	0.017
29.5	0.123	0.533	0.050	0.174	0.464	0.025	0.224	0.441	0.017

Table S27. Statistics used for the inter-group comparison between 3-year changes of depth-of-focus (ΔDOF) at each ε and 3-year changes of the axial length to corneal radius ratio (ΔAXL/CR). PCC, Pearson correlation coefficient; p, probability value; α, significance threshold adjusted using the Holm-Bonferroni method. * denotes p<α.

ε [deg]	LR			HR			MYO		
	PCC	p	α	PCC	p	α	PCC	p	α
-29.5	-0.266	0.172	0.025	-0.170	0.473	0.050	-0.450	0.107	0.017
-27.5	-0.168	0.394	0.050	-0.280	0.231	0.025	-0.376	0.185	0.017
-25.5	0.014	0.944	0.050	-0.365	0.114	0.025	-0.582	0.029	0.017
-23.5	-0.097	0.625	0.025	-0.278	0.235	0.017	-0.050	0.865	0.050
-21.5	-0.258	0.185	0.025	-0.343	0.139	0.017	0.232	0.424	0.050
-19.5	-0.263	0.176	0.025	-0.295	0.206	0.050	0.531	0.051	0.017
-17.5	-0.292	0.131	0.050	-0.421	0.064	0.025	0.564	0.036	0.017
-15.5	-0.398	0.036	0.017	-0.416	0.068	0.025	0.314	0.274	0.050
-13.5	-0.432	0.022	0.017	-0.410	0.073	0.050	0.596	0.024	0.025
-11.5	-0.389	0.041	0.017	-0.364	0.115	0.025	0.428	0.127	0.050
-9.5	-0.290	0.134	0.017	-0.329	0.156	0.025	0.114	0.698	0.050
-7.5	-0.376	0.049	0.017	-0.291	0.214	0.025	0.037	0.901	0.050
-5.5	-0.368	0.054	0.017	-0.391	0.088	0.025	0.177	0.546	0.050
-3.5	-0.435	0.021	0.017	-0.450	0.047	0.025	0.043	0.884	0.050
-1.5	-0.392	0.039	0.017	-0.402	0.079	0.025	0.129	0.660	0.050
0	-0.522	0.004*	0.017	-0.476	0.034	0.025	0.098	0.739	0.050
1.5	-0.371	0.052	0.017	-0.303	0.195	0.025	0.027	0.927	0.050
3.5	-0.276	0.155	0.025	-0.419	0.066	0.017	-0.012	0.968	0.050
5.5	-0.345	0.073	0.017	-0.337	0.146	0.025	0.165	0.572	0.050
7.5	-0.343	0.074	0.017	-0.178	0.452	0.050	0.238	0.412	0.025
9.5	-0.295	0.128	0.017	-0.191	0.420	0.050	0.346	0.226	0.025
21.5	-0.187	0.342	0.050	-0.327	0.160	0.025	0.531	0.051	0.017
23.5	-0.075	0.703	0.050	-0.129	0.586	0.025	0.664	0.010*	0.017
25.5	-0.058	0.769	0.025	0.065	0.785	0.050	0.512	0.061	0.017
27.5	-0.010	0.959	0.050	-0.024	0.921	0.025	0.520	0.057	0.017
29.5	0.031	0.876	0.050	-0.049	0.837	0.025	0.313	0.276	0.017

Table S28. Statistics used for the inter-group comparison between 3-year changes of maximum Strehl ratio (ΔMaxSR) at each ε and 3-year changes of the negative central spherocylindrical error (-ΔcSE). PCC, Pearson correlation coefficient; p, probability value; α, significance threshold adjusted using the Holm-Bonferroni method. * denotes p<α.

ε [deg]	LR			HR			MYO		
	PCC	p	α	PCC	p	α	PCC	p	α
-29.5	0.295	0.127	0.017	-0.044	0.854	0.050	-0.223	0.443	0.025
-27.5	0.133	0.500	0.050	-0.231	0.326	0.017	-0.243	0.402	0.025
-25.5	-0.025	0.899	0.050	-0.226	0.339	0.025	-0.298	0.300	0.017
-23.5	-0.141	0.475	0.050	-0.211	0.371	0.025	-0.585	0.028	0.017
-21.5	0.123	0.531	0.050	-0.195	0.411	0.025	-0.660	0.010*	0.017
-19.5	0.101	0.609	0.050	-0.200	0.397	0.025	-0.769	0.001*	0.017
-17.5	0.132	0.502	0.025	-0.076	0.751	0.050	-0.712	0.004*	0.017
-15.5	0.233	0.234	0.025	-0.010	0.966	0.050	-0.504	0.066	0.017
-13.5	0.283	0.145	0.025	0.003	0.990	0.050	-0.559	0.038	0.017
-11.5	0.253	0.194	0.025	0.167	0.481	0.050	-0.554	0.040	0.017
-9.5	0.265	0.173	0.017	0.171	0.472	0.050	-0.264	0.362	0.025
-7.5	0.420	0.026	0.017	0.068	0.775	0.025	-0.084	0.775	0.050
-5.5	0.489	0.008*	0.017	-0.048	0.839	0.025	0.029	0.921	0.050
-3.5	0.558	0.002*	0.017	-0.086	0.720	0.025	0.078	0.790	0.050
-1.5	0.489	0.008*	0.017	-0.179	0.450	0.025	0.117	0.691	0.050
0	0.554	0.002*	0.017	-0.208	0.378	0.025	-0.010	0.972	0.050
1.5	0.529	0.004*	0.017	-0.007	0.978	0.050	0.111	0.704	0.025
3.5	0.400	0.035	0.017	-0.157	0.508	0.025	0.148	0.615	0.050
5.5	0.371	0.052	0.017	-0.109	0.646	0.050	-0.139	0.635	0.025
7.5	0.415	0.028	0.017	-0.171	0.470	0.025	-0.165	0.573	0.050
9.5	0.152	0.440	0.050	-0.186	0.432	0.025	-0.244	0.400	0.017
21.5	-0.061	0.757	0.050	-0.385	0.093	0.017	-0.375	0.186	0.025
23.5	-0.141	0.475	0.050	-0.495	0.027	0.025	-0.800	5.9×10^{-4}*	0.017
25.5	-0.218	0.265	0.050	-0.262	0.264	0.025	-0.434	0.121	0.017
27.5	-0.054	0.783	0.050	-0.330	0.156	0.025	-0.457	0.100	0.017
29.5	-0.113	0.566	0.050	-0.417	0.067	0.017	-0.306	0.287	0.025

Table S29. Statistics used for the inter-group comparison between 3-year changes of maximum Strehl ratio (ΔMaxSR) at each ε and 3-year changes of the axial length to corneal radius ratio (ΔAXL/CR). PCC, Pearson correlation coefficient; p, probability value; α, significance threshold adjusted using the Holm-Bonferroni method. * denotes p<α.

ε [deg]	LR			HR			MYO		
	PCC	p	α	PCC	p	α	PCC	p	α
-29.5	0.101	0.609	0.025	0.482	0.031	0.017	0.122	0.678	0.050
-27.5	0.046	0.815	0.050	0.601	0.005*	0.017	0.089	0.762	0.025
-25.5	-0.058	0.768	0.025	0.529	0.016*	0.017	-0.041	0.890	0.050
-23.5	-0.050	0.801	0.050	0.468	0.038	0.017	-0.420	0.135	0.025
-21.5	0.091	0.647	0.050	0.556	0.011*	0.017	-0.476	0.085	0.025
-19.5	0.070	0.724	0.050	0.528	0.017*	0.025	-0.743	0.002*	0.017
-17.5	0.128	0.517	0.050	0.490	0.028	0.025	-0.847	1.3×10^{-4}*	0.017
-15.5	0.272	0.162	0.050	0.453	0.045	0.025	-0.635	0.015*	0.017
-13.5	0.424	0.025*	0.025	0.332	0.153	0.050	-0.756	0.002*	0.017
-11.5	0.351	0.067	0.025	0.366	0.113	0.050	-0.731	0.003*	0.017
-9.5	0.293	0.130	0.017	0.296	0.205	0.050	-0.393	0.164	0.025
-7.5	0.330	0.087	0.017	0.357	0.122	0.025	-0.211	0.468	0.050
-5.5	0.513	0.005*	0.017	0.435	0.055	0.025	-0.188	0.521	0.050
-3.5	0.509	0.006*	0.017	0.275	0.241	0.025	-0.032	0.914	0.050
-1.5	0.537	0.003*	0.017	0.227	0.337	0.025	-0.122	0.679	0.050
0	0.543	0.003*	0.017	0.184	0.437	0.025	-0.207	0.478	0.050
1.5	0.497	0.007*	0.017	0.190	0.423	0.025	-0.058	0.843	0.050
3.5	0.245	0.210	0.017	0.267	0.255	0.025	-0.008	0.978	0.050
5.5	0.468	0.012*	0.017	0.234	0.321	0.025	-0.193	0.509	0.050
7.5	0.480	0.010*	0.017	0.072	0.764	0.050	-0.319	0.266	0.025
9.5	0.339	0.078	0.017	0.033	0.891	0.050	-0.480	0.083	0.025
21.5	0.084	0.669	0.050	0.354	0.126	0.025	-0.533	0.050	0.017
23.5	0.122	0.537	0.050	0.264	0.260	0.025	-0.832	2.2×10^{-4}*	0.017
25.5	0.044	0.824	0.025	-0.021	0.930	0.050	-0.433	0.122	0.017
27.5	0.045	0.821	0.050	0.104	0.661	0.025	-0.516	0.059	0.017
29.5	-0.002	0.991	0.050	0.287	0.220	0.025	-0.423	0.131	0.017

Table S30. Statistics used for the inter-group comparison between 3-year changes of center of mass (ΔCOM) at each ε and 3-year changes of the negative central spherocylindrical error (-ΔcSE). PCC, Pearson correlation coefficient; p, probability value; α, significance threshold adjusted using the Holm-Bonferroni method. * denotes p<α.

ε [deg]	LR			HR			MYO		
	PCC	p	α	PCC	p	α	PCC	p	α
-29.5	0.112	0.571	0.025	0.025	0.917	0.050	-0.671	0.009*	0.017
-27.5	0.065	0.742	0.025	0.017	0.942	0.050	-0.644	0.013*	0.017
-25.5	-0.055	0.781	0.025	-0.038	0.874	0.050	-0.614	0.020	0.017
-23.5	-0.099	0.617	0.025	-0.101	0.672	0.050	-0.674	0.008*	0.017
-21.5	0.008	0.969	0.050	-0.125	0.598	0.025	-0.668	0.009*	0.017
-19.5	0.090	0.647	0.050	-0.124	0.603	0.025	-0.649	0.012*	0.017
-17.5	0.044	0.823	0.050	-0.169	0.477	0.025	-0.756	0.002*	0.017
-15.5	-0.102	0.604	0.050	-0.181	0.444	0.025	-0.616	0.019	0.017
-13.5	-0.069	0.728	0.050	-0.169	0.476	0.025	-0.329	0.251	0.017
-11.5	-0.067	0.735	0.050	-0.152	0.522	0.025	-0.421	0.134	0.017
-9.5	-0.056	0.775	0.050	-0.160	0.500	0.025	-0.389	0.169	0.017
-7.5	-0.042	0.832	0.050	-0.092	0.698	0.025	-0.140	0.633	0.017
-5.5	0.057	0.775	0.025	-0.063	0.792	0.050	-0.139	0.636	0.017
-3.5	-0.039	0.842	0.050	-0.073	0.759	0.025	-0.281	0.330	0.017
-1.5	0.044	0.824	0.025	-0.006	0.981	0.050	-0.373	0.189	0.017
0	0.204	0.298	0.017	-0.217	0.359	0.025	0.013	0.964	0.050
1.5	0.173	0.379	0.025	-0.079	0.742	0.050	-0.364	0.201	0.017
3.5	0.037	0.852	0.025	-0.080	0.738	0.017	-0.029	0.921	0.050
5.5	0.157	0.426	0.025	0.036	0.879	0.050	0.267	0.356	0.017
7.5	0.310	0.109	0.017	0.207	0.382	0.025	0.029	0.920	0.050
9.5	0.243	0.212	0.017	0.020	0.935	0.050	-0.047	0.874	0.025
21.5	-0.448	0.017	0.017	-0.068	0.777	0.050	0.343	0.230	0.025
23.5	-0.080	0.686	0.050	0.108	0.650	0.025	-0.275	0.341	0.017
25.5	0.017	0.932	0.025	-0.004	0.986	0.050	-0.374	0.188	0.017
27.5	0.086	0.663	0.025	-0.060	0.801	0.050	-0.408	0.148	0.017
29.5	0.095	0.631	0.050	-0.163	0.492	0.025	-0.393	0.164	0.017

Table S31. Statistics used for the inter-group comparison between 3-year changes of center of mass (ΔCOM) at each ε and 3-year changes of the axial length to corneal radius ratio (ΔAXL/CR). PCC, Pearson correlation coefficient; p, probability value; α, significance threshold adjusted using the Holm-Bonferroni method. * denotes p<α.

ε [deg]	LR			HR			MYO		
	PCC	p	α	PCC	p	α	PCC	p	α
-29.5	-0.262	0.178	0.050	-0.484	0.030	0.017	-0.560	0.037	0.025
-27.5	-0.238	0.222	0.050	-0.527	0.017	0.017	-0.612	0.020	0.025
-25.5	-0.322	0.095	0.050	-0.559	0.010*	0.017	-0.657	0.011*	0.025
-23.5	-0.262	0.178	0.050	-0.523	0.018*	0.025	-0.700	0.005*	0.017
-21.5	-0.275	0.157	0.050	-0.478	0.033	0.025	-0.731	0.003*	0.017
-19.5	-0.127	0.519	0.050	-0.484	0.030	0.025	-0.727	0.003*	0.017
-17.5	-0.159	0.418	0.050	-0.462	0.040	0.025	-0.798	6.2×10^{-4}*	0.017
-15.5	-0.272	0.162	0.050	-0.426	0.061	0.025	-0.587	0.027	0.017
-13.5	-0.221	0.258	0.025	-0.359	0.120	0.017	-0.272	0.346	0.050
-11.5	-0.160	0.415	0.050	-0.306	0.189	0.025	-0.489	0.076	0.017
-9.5	-0.240	0.219	0.025	-0.244	0.300	0.050	-0.489	0.076	0.017
-7.5	-0.288	0.137	0.025	-0.109	0.648	0.050	-0.428	0.127	0.017
-5.5	-0.318	0.099	0.025	-0.225	0.340	0.050	-0.470	0.090	0.017
-3.5	-0.322	0.094	0.017	-0.253	0.282	0.050	-0.411	0.145	0.025
-1.5	-0.262	0.178	0.017	-0.181	0.445	0.025	-0.152	0.604	0.050
0	0.082	0.680	0.025	-0.137	0.564	0.017	-0.001	0.998	0.050
1.5	-0.109	0.582	0.025	-0.039	0.870	0.050	-0.174	0.553	0.017
3.5	-0.137	0.485	0.017	-0.111	0.642	0.025	-0.040	0.893	0.050
5.5	-0.114	0.564	0.017	-0.110	0.645	0.025	0.103	0.725	0.050
7.5	0.052	0.791	0.025	-0.252	0.283	0.017	0.001	0.998	0.050
9.5	0.105	0.595	0.025	-0.189	0.424	0.017	0.121	0.681	0.050
21.5	-0.161	0.414	0.025	-0.133	0.576	0.050	0.394	0.164	0.017
23.5	-0.010	0.958	0.050	-0.186	0.432	0.017	-0.216	0.458	0.025
25.5	-0.022	0.911	0.050	-0.166	0.485	0.025	-0.474	0.087	0.017
27.5	0.023	0.906	0.050	-0.053	0.824	0.025	-0.511	0.062	0.017
29.5	-0.003	0.988	0.050	-0.065	0.786	0.025	-0.497	0.071	0.017

Table S32. Statistics used for the inter-group comparison between 3-year changes of logarithmic optical anisotropy ($\Delta Log_{10}(OAn)$) at each ε and 3-year changes of the negative central spherocylindrical error (-ΔcSE). PCC, Pearson correlation coefficient; p, probability value; α, significance threshold adjusted using the Holm-Bonferroni method. * denotes p<α.

ε [deg]	LR			HR			MYO		
	PCC	p	α	PCC	p	α	PCC	p	α
-29.5	0.084	0.670	0.025	-0.050	0.834	0.050	0.534	0.049	0.017
-27.5	0.034	0.865	0.050	-0.096	0.686	0.025	0.491	0.075	0.017
-25.5	0.069	0.728	0.025	-0.017	0.943	0.050	0.368	0.195	0.017
-23.5	0.122	0.537	0.025	-0.048	0.840	0.050	0.372	0.190	0.017
-21.5	0.072	0.716	0.025	-0.022	0.927	0.050	0.360	0.207	0.017
-19.5	-0.037	0.852	0.050	0.180	0.448	0.025	0.370	0.192	0.017
-17.5	-0.192	0.327	0.025	0.079	0.742	0.050	0.321	0.263	0.017
-15.5	-0.109	0.581	0.050	0.216	0.360	0.017	0.216	0.458	0.025
-13.5	-0.118	0.551	0.025	0.177	0.455	0.017	0.067	0.819	0.050
-11.5	-0.097	0.622	0.017	0.040	0.867	0.050	0.078	0.792	0.025
-9.5	0.183	0.350	0.017	0.072	0.764	0.050	-0.138	0.637	0.025
-7.5	0.248	0.204	0.017	0.022	0.927	0.025	0.002	0.995	0.050
-5.5	0.409	0.031	0.017	0.012	0.959	0.025	0.006	0.983	0.050
-3.5	0.383	0.044	0.017	-0.142	0.549	0.025	-0.072	0.808	0.050
-1.5	0.045	0.819	0.017	-0.046	0.848	0.025	-0.054	0.855	0.050
0	0.163	0.406	0.017	-0.133	0.577	0.050	-0.236	0.416	0.025
1.5	0.004	0.985	0.050	-0.111	0.643	0.017	-0.018	0.951	0.025
3.5	0.005	0.979	0.050	-0.047	0.844	0.025	-0.168	0.565	0.017
5.5	0.232	0.235	0.017	0.009	0.970	0.050	-0.263	0.363	0.025
7.5	0.014	0.942	0.050	0.134	0.572	0.025	-0.264	0.362	0.017
9.5	0.126	0.522	0.025	0.114	0.632	0.050	-0.221	0.448	0.017
21.5	-0.065	0.742	0.050	0.336	0.147	0.017	0.152	0.604	0.025
23.5	-0.027	0.890	0.050	0.329	0.156	0.017	0.048	0.871	0.025
25.5	0.314	0.104	0.017	0.269	0.252	0.025	-0.123	0.676	0.050
27.5	0.311	0.108	0.017	0.287	0.220	0.025	-0.175	0.550	0.050
29.5	0.256	0.189	0.025	0.315	0.176	0.017	-0.159	0.588	0.050

Table S33. Statistics used for the inter-group comparison between 3-year changes of logarithmic optical anisotropy ($\Delta Log_{10}(OAn)$) at each ε and 3-year changes of the axial length to corneal radius ratio (ΔAXL/CR). PCC, Pearson correlation coefficient; p, probability value; α, significance threshold adjusted using the Holm-Bonferroni method. * denotes p<α.

ε [deg]	LR			HR			MYO		
	PCC	p	α	PCC	p	α	PCC	p	α
-29.5	0.081	0.681	0.050	0.521	0.018	0.017	0.548	0.042	0.025
-27.5	0.108	0.584	0.050	0.426	0.061	0.025	0.575	0.031	0.017
-25.5	0.089	0.651	0.050	0.390	0.090	0.025	0.575	0.031	0.017
-23.5	0.031	0.875	0.050	0.299	0.200	0.025	0.521	0.056	0.017
-21.5	-0.021	0.916	0.050	0.371	0.107	0.025	0.491	0.074	0.017
-19.5	-0.077	0.696	0.050	0.305	0.191	0.025	0.569	0.034	0.017
-17.5	-0.287	0.139	0.050	0.471	0.036	0.017	0.449	0.107	0.025
-15.5	-0.270	0.164	0.025	0.508	0.022	0.017	0.384	0.175	0.050
-13.5	-0.391	0.040	0.017	0.395	0.085	0.025	0.212	0.467	0.050
-11.5	-0.398	0.036	0.025	0.521	0.019	0.017	0.190	0.515	0.050
-9.5	-0.136	0.489	0.025	0.343	0.138	0.017	0.044	0.880	0.050
-7.5	-0.003	0.986	0.050	0.277	0.236	0.017	0.217	0.457	0.025
-5.5	0.081	0.682	0.050	0.197	0.405	0.025	0.254	0.382	0.017
-3.5	0.084	0.670	0.050	0.114	0.634	0.025	0.170	0.562	0.017
-1.5	-0.218	0.266	0.017	0.080	0.738	0.050	0.131	0.655	0.025
0	-0.034	0.863	0.025	-0.097	0.685	0.017	0.035	0.906	0.050
1.5	-0.127	0.521	0.025	-0.179	0.450	0.017	0.147	0.615	0.050
3.5	-0.177	0.368	0.017	-0.070	0.769	0.025	0.049	0.868	0.050
5.5	-0.042	0.833	0.025	-0.133	0.576	0.017	-0.037	0.900	0.050
7.5	-0.105	0.594	0.025	-0.312	0.181	0.017	-0.092	0.753	0.050
9.5	-0.005	0.978	0.050	-0.637	0.003*	0.017	-0.083	0.779	0.025
21.5	-0.170	0.386	0.025	-0.361	0.118	0.017	0.102	0.730	0.050
23.5	-0.209	0.285	0.025	-0.493	0.027	0.017	0.165	0.572	0.050
25.5	0.070	0.722	0.025	-0.370	0.109	0.017	0.098	0.739	0.050
27.5	0.066	0.738	0.025	-0.361	0.118	0.017	0.094	0.748	0.050
29.5	0.034	0.866	0.050	-0.126	0.596	0.017	0.062	0.833	0.025

References

1. F. A. Vera-Diaz, A. Jnawali, A. Panorgias, P. J. Bex, and K. L. Kerber, "Baseline metrics that may predict future myopia in young children," Ophthalmic Physiol. Opt. **43**, 466–481 (2023).
2. F. A. Vera-Diaz, D. Dhungel, A. McCullough, K. L. Kerber, and P. J. Bex, "Longitudinal measures of peripheral optical quality in young children," Ophthalmic Physiol. Opt. **45**, 550–564 (2025).
